# Reduced expression of the psychiatric risk gene DLG2 (PSD93) impairs hippocampal synaptic integration and plasticity

**DOI:** 10.1038/s41386-022-01277-6

**Published:** 2022-02-03

**Authors:** Simonas Griesius, Cian O’Donnell, Sophie Waldron, Kerrie L. Thomas, Dominic M. Dwyer, Lawrence S. Wilkinson, Jeremy Hall, Emma S. J. Robinson, Jack R. Mellor

**Affiliations:** 1 Centre for Synaptic Plasticity, School of Physiology, Pharmacology and Neuroscience, University of Bristol, University Walk, Bristol, BS8 1TD UK; 2 Computational Neuroscience Unit, School of Computer Science, Electrical and Electronic Engineering, and Engineering Mathematics, University of Bristol, Bristol, BS8 1UB UK; 3 Neuroscience and Mental Health Research Institute, Cardiff, CF24 4HQ UK; 4 School of Psychology, Cardiff, CF24 4HQ UK; 5 School of Medicine, Cardiff, CF24 4HQ UK; 6 MRC Centre for Neuropsychiatric Genetics and Genomics, Cardiff, CF24 4HQ UK

**Keywords:** Cellular neuroscience, Long-term potentiation, Schizophrenia, Risk factors

## Abstract

Copy number variants indicating loss of function in the *DLG2* gene have been associated with markedly increased risk for schizophrenia, autism spectrum disorder, and intellectual disability. *DLG2* encodes the postsynaptic scaffolding protein DLG2 (PSD93) that interacts with NMDA receptors, potassium channels, and cytoskeletal regulators but the net impact of these interactions on synaptic plasticity, likely underpinning cognitive impairments associated with these conditions, remains unclear. Here, hippocampal CA1 neuronal excitability and synaptic function were investigated in a novel clinically relevant heterozygous *Dlg2*+*/−* rat model using ex vivo patch-clamp electrophysiology, pharmacology, and computational modelling. *Dlg2*+*/−* rats had reduced supra-linear dendritic integration of synaptic inputs resulting in impaired associative long-term potentiation. This impairment was not caused by a change in synaptic input since NMDA receptor-mediated synaptic currents were, conversely, increased and AMPA receptor-mediated currents were unaffected. Instead, the impairment in associative long-term potentiation resulted from an increase in potassium channel function leading to a decrease in input resistance, which reduced supra-linear dendritic integration. Enhancement of dendritic excitability by blockade of potassium channels or activation of muscarinic M1 receptors with selective allosteric agonist 77-LH-28-1 reduced the threshold for dendritic integration and 77-LH-28-1 rescued the associative long-term potentiation impairment in the *Dlg2*+*/−* rats. These findings demonstrate a biological phenotype that can be reversed by compound classes used clinically, such as muscarinic M1 receptor agonists, and is therefore a potential target for therapeutic intervention.

## Introduction

Genetic variations at the *DLG2* gene locus are linked to multiple psychiatric disorders including schizophrenia [1, 2], bipolar [3, 4], autism spectrum [5–7], attention deficit hyperactivity [8], intellectual disability [9, 10], and Parkinson’s disease [11, 12]. This clinical evidence indicates the significance of DLG2 in the aetiology of psychopathologies common to a broad range of disorders and suggests core underlying mechanisms and biological phenotypes. Many of the genetic variations are predicted to produce a loss of function for DLG2 in one copy of the gene but the resulting changes in neuronal function are poorly understood [13–16].

DLG2 is a member of a family of membrane-associated guanylate kinase (MAGUK) proteins enriched at synaptic locations that encodes the scaffolding protein PSD93 (also referred to as DLG2 or Chapsyn-110). DLG2 interacts directly with a number of other proteins in the postsynaptic density of excitatory synapses, such as NMDA receptor (NMDAR) subunit GluN2B [17–20], AMPA receptor auxiliary subunit stargazin [21], potassium channels Kir2.3 [22], Kir2.2 [23] and Kv1.4 [24], as well as proteins involved in potassium channel palmitoylation, cell adhesion, microtubule assembly, and cell signalling, palmitoyltransferase ZDHHC14 [25], neuroligin1-3 [17], Fyn [26, 27], ERK2 [28], GKAP [29], and MAP1A [30]. Uniquely to the MAGUK family, DLG2 is targeted to the axon initial segment where it regulates neuronal excitability via its interactions with potassium channels [25, 31]. At a functional level, homozygous *Dlg2−/−* knockout mice have altered glutamatergic synapse function [32–35] and impaired long-term potentiation (LTP) in the hippocampus [33]. These synaptic perturbations could underlie the common cognitive psychopathologies of the psychiatric disorders associated with *DLG2*. Indeed, *Dlg2−/−* mice have been shown to have impaired performance in the object-location paired associates learning task [36]. Homozygous *Dlg2−/−* mice also exhibit increased grooming behaviour [35] and altered social interaction but without consistent effects in negative valence tasks such as the open field test [35, 37].

Impaired synaptic plasticity resulting from the loss of DLG2 is a potential biological phenotype underpinning trans-diagnostic cognitive psychopathologies but the mechanism by which reduced DLG2 expression leads to impaired synaptic plasticity is unclear. Furthermore, mechanistic understanding for the impact of DLG2 loss may reveal new targets for therapeutic intervention. Most animal models for DLG2 loss have employed full knockouts of the gene but these do not accurately represent the heterozygous nature of DLG2 genetic variants in patient populations and potentially engage compensatory expression by other MAGUK proteins [32, 38] that is not present in heterozygous reduced gene dosage models (Supplementary Fig. S3). Therefore, here we investigate the combined impact of low gene dosage DLG2 on synaptic function, neuronal excitability and morphology using a novel CRISPR-Cas9 engineered heterozygous *Dlg2*+*/−* (het) rat model to understand the interactions that lead to impaired synaptic plasticity and cognitive function.

## Materials and methods

### Animals and husbandry

All procedures were carried out under local institutional guidelines, approved by the University of Bristol Animal Welfare and Ethical Review Board, and in accordance with the UK Animals (Scientific procedures) Act 1986. The experiments employed a novel *Dlg2*+*/−* heterozygous rat model generated on a Hooded Long Evans background using CRISPR-Cas9 genomic engineering that targeted a 7 bp deletion to exon 5 of the rat *Dlg2* gene, resulting in a downstream frame shift in exon 6 and the production of a premature stop codon that led to a reduction in Dlg2 protein levels in the hippocampus (Supplementary Figs. S1–3). Full details of the creation, quality control and off-target assessment of the *Dlg2*+*/−* model can be found in Supplementary information. Male *Dlg2*+*/−* rats were bred with wild type (wt) female rats, generating mixed litters of *Dlg2*+*/−* and wt littermate offspring. The *Dlg2*+*/−* animals were viable and showed no signs of ill health, with normal litter sizes containing the expected Mendelian ratio of positive to wt genotypes and normal sex ratios, there were no effects in survival of the *Dlg2*+*/−* rats to adulthood and no effects on general morbidity, including fertility, or mortality throughout the lifespan. Further details of animal husbandry, breeding strategy and viability are described in Supplementary information. Approximately equal numbers of each sex rats aged P50-75 were used, with experimenter blind to genotype during experiments and data analysis.

Methods on brain slice preparation, electrophysiology, protein quantification and computational modelling are in the supplementary information.

### Statistical analysis

3-way and 2-way ANOVA, 3-way repeated measures ANOVA, Komolgorov–Smirnov test, as well as paired and unpaired t-tests were used as appropriate, with full statistical results available in Supplement 2. Genotype, sex, and dorsal-ventral aspects of the hippocampus, as well as repeated measurements, were factored into all analyses, as appropriate. Genotype was viewed as the primary output factor shown in the figures. No genotype-sex and only one genotype-aspect interactions were found indicating limited impact of sex and aspect on the primary genotype factor results but where effects of other factors were found, the data are presented in Supplement 1 (Supplementary Figs. S8–16, S18 and 19). Inclusion of animals in the analysis of a subset of experiments using multi-level general linear mixed modelling did not affect the statistical results indicating that the major source of variability arose between cells rather than animals. Therefore, cell was defined as the experimental unit and we report numbers of cells and animals in figure legends. α = 0.05 was applied for all tests, except the Kolmogorov–Smirnov test where α = 0.01 was applied. The degrees of freedom, *F*, and *P* values are presented in the text, figures, and Supplement 2.

## Results

*Dlg2*+*/−* heterozygous knockout rats were generated by CRISPR-Cas9 targeting of the *Dlg2* gene (Supplementary information). In this model, DLG2 protein expression levels were reduced by ~50% in hippocampus without effects on expression of other components of the postsynaptic density, including the closely related MAGUK DLG4 (PSD95) and the GluN1 NMDAR subunit (Supplementary Fig. S3). The specific reduction in DLG2 protein expression was replicated in tissue from prefrontal cortex, posterior cortex and cerebellum and mirrored by a ~50% reduction of *dlg2* mRNA expression, without any change in *dlg1*, *dlg3* or *dlg4* mRNA [39].

### Associative LTP

The learning of novel representations in CA1 is thought to arise from the dendritic integration of spatiotemporally coherent inputs from the entorhinal cortex (via the temporoammonic (TA) pathway) and CA3 (via the Schaffer collateral (SC) pathway) that can summate supra-linearly to drive associative LTP (aLTP) [40–47]. LTP in CA1 of hippocampus is impaired in homozygous *Dlg2*−/− mice [33] but the interpretation of these results is complicated by the potential for compensation by other MAGUK proteins [32, 38]. The heterozygous *Dlg2*+/− rat offers the opportunity to test whether LTP is impaired in the absence of any MAGUK compensation.

**Fig. 1:**
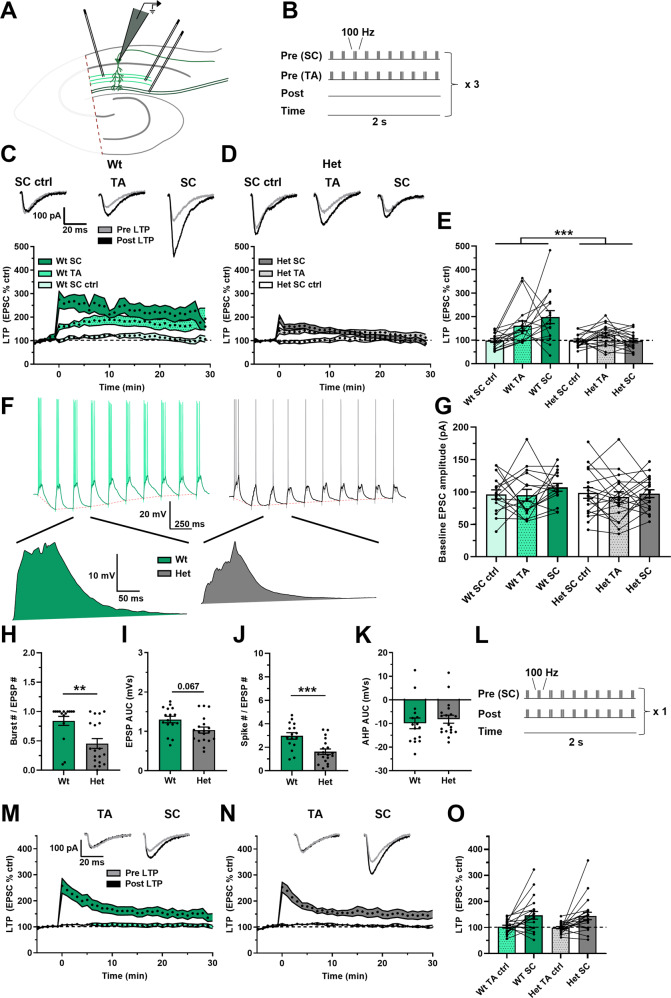
Attenuated aLTP in the *Dlg2*+*/−* hets despite normal TBS LTP. **A** Schematic representation of the hippocampal slice recording setup, with the CA3 removed and stimulating electrodes in two separate areas of the stratum radiatum and in the stratum lacunosum moleculare. **B** aLTP induction protocol, where one SC pathway and one TA pathway were tested and where the second SC pathway acted as a negative control. There was no induced somatic depolarisation. This induction protocol was repeated thrice at an interval of 10 s. aLTP over time in wts (**C**) and *Dlg2*+*/−* hets (**D**). Example traces pre- and post-induction are displayed for the wt and het groups above their corresponding plots of LTP over time. **E** aLTP at the 25–30 min mark post induction across genotype and pathway (3-way repeated-measures ANOVA: pathway effect: *F*_2, 54_ = 7.300, *P* = 0.002. Genotype main effect: *F*_1, 27_ = 15.687, *P* < 0.001. Genotype x pathway interaction: *F*_2, 54_ = 7.376, *P* = 0.001). **F** Example traces of LTP induction, with example EPSPs following *post hoc* spike truncation. **G** Baseline EPSC amplitude across genotype (3-way repeated-measures ANOVA: pathway effect: *F*_2, 54_ = 1.227, *P* = 0.301. Genotype main effect: *F*_1, 27_ = 0.553, *P* = 0.463. Genotype x pathway interaction: *F*_2, 54_ = 0.344, *P* = 0.711). Burst number (3-way ANOVA: genotype main effect: *F*_1, 27_ = 10.407, *P* = 0.003) (**H**), EPSP AUC (3-way ANOVA: genotype main effect: *F*_1, 27_ = 3.63, *P* = 0.067) (**I**), total spike number (3-way ANOVA: genotype main effect: *F*_1, 27_ = 15.877, *P* < 0.001) (**J**), and afterhyperpolarisation AUC (3-way ANOVA: genotype main effect: *F*_1, 27_ = 0.036, *P* = 0.85) (**K**) across genotype during LTP induction. Hets: 19 cells, 9 animals and wts: 16 cells, 8 animals. **L** Theta burst LTP induction protocol, where the SC pathway was paired with somatic depolarisation and where the TA pathway acted as a negative control. Theta burst LTP over time in wts (**M**) and *Dlg2*+*/−* hets (**N**). **O** Theta burst LTP at the 25–30 min mark post induction across genotype (3-way repeated-measures ANOVA: pathway effect: *F*_1, 33_ = 18.979, *P* < 0.001. Genotype main effect: *F*_1, 33_ = 0.04, *P* = 0.843. Genotype x pathway interaction: *F*_1, 33_ = 0.004, *P* = 0.950). Hets: 21 cells, 9 animals and wts: 20 cells, 10 animals. Summary values depicted as mean ± SEM. *P < 0.05, **P < 0.01, ***P < 0.001 (3-way ANOVA between subject effect).

aLTP was assessed in the CA1 region of hippocampal slices by stimulating the SC and TA pathways simultaneously with a theta burst stimulation pattern whilst recording from CA1 pyramidal neurons (Supplementary Fig. 1A, B). An additional independent SC pathway was also stimulated as a negative control and a pathway check was done to confirm pathway independence (Supplementary Fig. S4). The induction protocol resulted in robust aLTP in the wts but reduced aLTP in the *Dlg2*+*/−* hets in both SC and TA test pathways (Fig. 1C–E). During induction the number of elicited action potential bursts and single spikes was reduced in the *Dlg2*+*/−* hets, despite baseline EPSC amplitudes being unchanged indicating that all neurons received similar inputs, but there was a trend suggesting reduced overall depolarisation in response to synaptic stimulation (Fig. 1F–J). Both spike number and depolarisation during induction correlated with LTP in the SC pathway but not the TA pathway (Supplementary Fig. S5). There was no effect of genotype on the after-hyperpolarisation (Fig. 1K). This suggests that in the *Dlg2*+*/−* hets, the integration of synaptic inputs from the SC and TA pathways is impaired, reducing dendritic depolarisation and action potential spiking which are the drivers of aLTP.

To test the necessity of action potentials for aLTP, a paired theta burst LTP induction protocol was used, where action potentials were driven by somatic current injection to bypass dendritic integration, and spikes were paired with simultaneous SC pathway stimulation (Fig. 1L). Under these conditions, robust LTP was induced in the SC pathway in both genotypes, with the TA pathway acting as negative control (Fig. 1M–O). Similarly, when aLTP was tested using baseline EPCSs doubled in amplitude, maximal LTP was induced and there was no effect of genotype (Supplementary Figs. S6 and 7). As expected, aLTP was greater in ventral slices whereas theta burst LTP was greater in dorsal slices (Supplementary Figs. S8 and 9). This indicates that the hets are fundamentally able to undergo LTP but their ability to integrate inputs is impaired.

### Synaptic integration

**Fig. 2:**
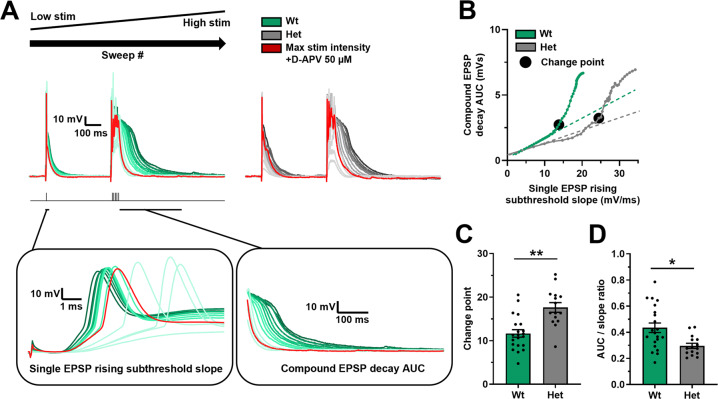
Increased threshold for supra-linear dendritic integration in the *Dlg2*+*/−* hets in the SC pathway. **A** Example traces depicting a single EPSP followed by a compound EPSP at increasing stimulation intensities (light to dark) over consecutive recording sweeps. The red trace represents the response at maximal stimulation intensity in the presence of 50 µM D-APV. The left inset depicts a zoomed-in view of the single EPSP, where rising subthreshold slope was measured. The right inset depicts a zoomed-in view of the compound EPSP decay, where the AUC was measured. **B** Example relationships between the single EPSP rising subthreshold slope and the compound EPSP decay AUC across genotype, with the change points marked by black circles. Change point (EPSP rising subthreshold slope mV/mS) (3-way ANOVA: genotype main effect: *F*_1, 34_ = 12.625, *P* = 0.001) (**C**) and AUC/slope ratio (3-way ANOVA: genotype main effect: *F*_1, 34_ = 8.003, *P* = 0.009) (**D**) across genotype. Hets: 15 cells, 9 animals and wts: 19 cells, 12 animals. Summary values depicted as mean ± SEM. *P < 0.05, **P < 0.01, ***P < 0.001 (3-way ANOVA between subject effect).

To directly test synaptic integration, the number of activated synapses required to generate supra-linear summation of EPSPs across multiple dendrites was assessed, which is a measure of the ability for synapses to integrate across the dendritic arbor [40, 42, 47] and allowed comparison of synaptic integration between genotypes. To activate increasing numbers of synapses, the SC pathway was stimulated with increasing intensity to activate synapses with a random spatial distribution across the proximal and basal dendritic arbor. The number of activated synapses was measured by the slope of a single EPSP, and the integration of synapses assessed by the amplitude and duration of a compound summated EPSP (area under the curve – AUC) elicited by repetitive high frequency synaptic stimulation (Fig. 2A). As stimulation intensity was increased the number of active synapses increased in a linear relationship with the amplitude and durations of the summated compound EPSP until a “change point” was reached (see methods) after which the relationship became supra-linear because the duration of the compound EPSP increased (Fig. 2B), indicative of activation of regenerative or plateau potentials within the dendrites [40, 42, 47]. The inhibition of these regenerative potentials by D-APV demonstrated their dependence on NMDAR activation (Fig. 2A, B). The change point was increased in the *Dlg2*+*/−* hets (Fig. 2B, C), indicating that het neurons required more synaptic inputs to undergo the transition to supra-linear integration. Additionally, the maximum duration of the compound EPSP as a ratio to the corresponding slope of the single EPSP was reduced in the *Dlg2*+*/−* hets (Fig. 2D). This again indicates that *Dlg2*+*/−* hets require more synaptic input to integrate dendritic inputs and produce the supra-linear regenerative potentials important for aLTP.

### NMDAR currents

**Fig. 3:**
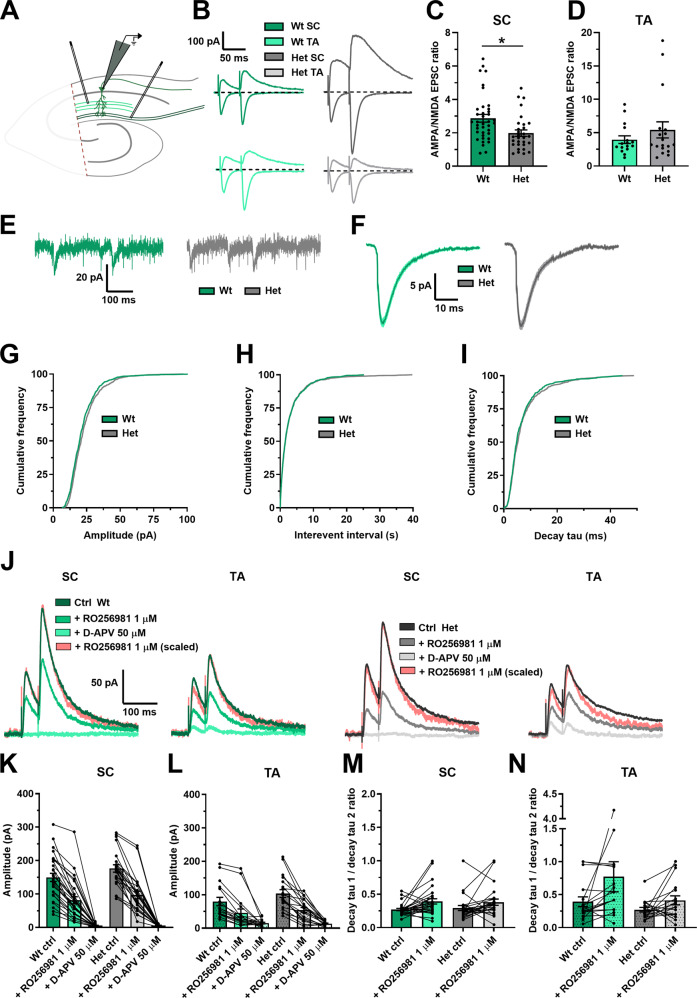
Glutamatergic function is altered in the *Dlg2*+*/−* hets due to an increase in NMDAR current, with no change in AMPAR current or GluN2b subunit expression. **A** Schematic representation of the hippocampal slice recording setup, with the CA3 removed and stimulating electrodes in the stratum radiatum and in the stratum lacunosum moleculare. **B** AMPA/NMDA EPSC ratio example traces. The primarily AMPAR-mediated EPSCs were recorded at a holding potential of −70 mV, whilst the AMPA- and NMDA-mediated EPSCs were recorded at a holding potential of 40 mV. The AMPA/NMDA ratio was derived by dividing the peak EPSC amplitude at −70 mV by the EPSC amplitude 45 ms after stimulation at 40 mV. AMPA/NMDA EPSC ratio across genotype in the SC (3-way ANOVA: genotype main effect: *F*_1, 68_ = 4.791, *P* = 0.033) (**C**) and the TA (3-way ANOVA: genotype main effect: *F*_1, 34_ = 0.583, *P* = 0.452) (**D**) pathways. Hets: 30 cells, 18 animals and wts: 38 cells, 19 animals for the SC data set and hets: 18 cells, 9 animals and wts: 16 cells, 8 animals for the TA data set. Example mEPSC traces (**E**) and mean mEPSCs (**F**) across genotype. Cumulative frequency plots of amplitude (Kolmogorov–Smirnov Test: *P* = 0.028) (**G**), interevent interval (Kolmogorov–Smirnov Test: *P* = 0.999) (**H**), and decay tau (Kolmogorov–Smirnov Test: *P* = 0.067) (**I**). Hets: 25 cells, 9 animals and wts: 21 cells, 5 animals. **J** GluN2b example EPSC traces. The traces following RO256981 1 µM administration were also peak scaled to better illustrate changes in decay kinetics. EPSC amplitude in the SC (3-way repeated-measures ANOVA: drug main effect: *F*_2, 88_ = 260.603, *P* < 0.001. Genotype x drug interaction: *F*_2, 88_ = 2.952, *P* = 0.057) (**K**) and TA (3-way repeated-measures ANOVA: drug main effect: *F*_2, 56_ = 42.076, *P* < 0.001. Genotype x drug interaction: *F*_2, 56_ = 1.738, *P* = 0.185) (**L**) pathways. Decay tau 1/decay tau 2 ratio in SC (3-way repeated-measures ANOVA: drug main effect: *F*_1, 45_ = 9.715, *P* = 0.003. Genotype x drug interaction: *F*_1, 45_ = 0.272, *P* = 0.605) (**M**) and TA (3-way repeated-measures ANOVA: drug main effect: *F*_1, 27_ = 3.410, *P* = 0.076. Genotype x drug interaction: *F*_1, 27_ = 0.831, *P* = 0.370) (**N**) pathways. Hets: 24 cells, 6 animals and wts: 29 cells, 8 animals for the SC data set and hets: 19 cells, 6 animals and wts: 18 cells, 8 animals for the TA data set. Summary values depicted as mean ± SEM. *P < 0.05, **P < 0.01, ***P < 0.001 (3-way ANOVA between subject effect).

Synaptic integration is driven by NMDARs and protein-protein interaction studies have reported DLG2 to interact directly with NMDAR subunits [17–19] and with AMPAR indirectly [21]. Further, DLG2 has also been shown to affect glutamatergic function in homozygous *Dlg2−/−* models, albeit with variable results in AMPA/NMDA ratio [32–34, 48, 49]. To investigate whether glutamatergic function was affected in *Dlg2*+*/−* rats and whether this might explain the impairment in synaptic integration and aLTP, the AMPA/NMDA ratio was measured in CA1 pyramidal neurons (Fig. 3A). *Dlg2*+*/−* hets had a reduced AMPA/NMDA ratio in the SC pathway, with no effect in the TA pathway (Fig. 3B–D). AMPAR-mediated miniature excitatory postsynaptic currents (mEPSCs, Supplementary Fig. S10) resulting from the activity of single synapses were recorded to probe whether the reduction in AMPA/NMDA ratio resulted from a reduction in AMPA, an increase in NMDA, or a combination of the two. The slow kinetics and small amplitude of NMDAR-mediated mEPSCs make them difficult to detect accurately. AMPAR-mediated mEPSCs from synapses on proximal dendrites are more detectable than those from more distal synapses due to signal attenuation and therefore recorded mEPSCs will arise from the most proximal synapses [50]. There was no difference in the distributions of mEPSC amplitude, interevent interval, or decay tau across genotype (Fig. 3E–I). Paired-pulse facilitation, measured in the AMPA/NMDA ratio experiment, was also not different across genotype in either pathway (Supplementary Fig. S11). Together, these results show no change in postsynaptic AMPAR function and presynaptic glutamate release probability in the SC pathway. It follows that the AMPA/NMDA ratio effect in the SC pathway was due to an increase in NMDAR function. This could result from either an increase in NMDAR number or a change in subunit composition between GluN2A and GluN2B. To test subunit composition, NMDAR currents were isolated (Fig. 3J) and the selective GluN2B negative allosteric modulator RO256981 was applied. RO256981 decreased EPSC amplitude (Fig. 3J–L) and increased the EPSC decay time in both the SC and the TA pathways (Fig. 3M, N). There was a trend toward a genotype x drug interaction in the EPSC amplitude measurement in the SC pathway but no genotype x drug interaction in the EPSC decay kinetics. Together, these results show similar NMDAR subunit composition across genotype and therefore the enhancement in synaptic NMDAR function likely arises from increased receptor numbers at SC synapses, despite overall neuronal receptor expression remaining constant. Enhanced synaptic NMDAR function is expected to increase synaptic integration and therefore cannot explain the observed decrease in integration.

### Input resistance

Reduced synaptic integration in dendrites could arise from multiple mechanisms. Based on previous findings in CA1 pyramidal neurons the three most likely are: i) Reduced expression of hyperpolarisation-activated cyclic nucleotide-gated (HCN) channels that regulate neuronal excitability and contribute to dendritic integration [51–53], ii) Increased expression of small conductance calcium-activated potassium (SK) channels that inhibit NMDARs at synapses, reducing dendritic integration and LTP [54–56], iii) Reduced input resistance by increased potassium channel expression particularly in dendritic locations to reduce dendritic integration and LTP [42, 47, 57–61]. Each of these mechanisms was directly tested.

Pharmacological blockade of HCN channels with ZD7288 produced robust effects on neuronal excitability (including spiking, sag, and input resistance) but there were no differential effects across genotype (Supplementary Fig. S12). There were also no genotype-specific effects on cellular resonance or impedance that are directly dependent on HCN channels [62–66] (Supplementary Fig. S13) with some effects of sex and aspect (Supplementary Figs. S14,15,17,18). As previously described, the SK channel blocker apamin produced an increase in EPSP duration in the SC and TA pathways (Supplementary Fig. S12) indicating increased NMDAR activation during synaptic stimulation [54–56]. However, the regulation of synaptic NMDAR function by SK channels was similar between genotypes indicating no change in SK channel expression. Therefore, differential HCN or SK channel function is unlikely to explain the difference in synaptic integration between genotypes.

**Fig. 4:**
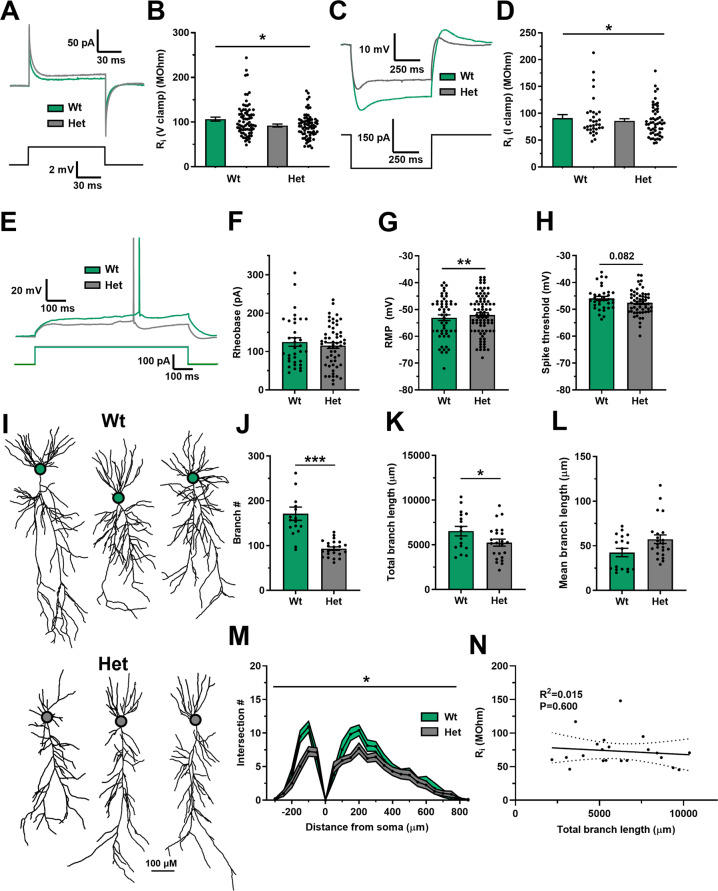
Reduced input resistance and dendritic arborisation in the *Dlg2*+*/−* hets. **A** Current traces in response to a 2 mV voltage step for wt and het. **B** Input resistance (V clamp) across genotype (3-way ANOVA: genotype main effect: *F*_1, 146_ = 5.698, *P* = 0.018). Hets: 73 cells, 28 animals and wts: 73 cells, 31 animals. **C** Voltage traces in response to a −150 pA current step across genotype. **D** Input resistance (I clamp) across genotype (3-way ANOVA: genotype main effect: *F*_1, 78_ = 4.209, *P* = 0.044). The sets of cells in panels **C** and **D** are separate and represent independent analyses. **E** Voltage deflections in response to a current step of equal size across genotype. Rheobase (3-way ANOVA: genotype main effect: *F*_1, 90_ = 0.011, *P* = 0.916) (**F**), resting membrane potential (RMP) (3-way ANOVA: genotype main effect: *F*_1, 136_ = 7.075, *P* = 0.009) (**G**), and spike threshold (3-way ANOVA: genotype main effect: *F*_1, 89_ = 3.105, *P* = 0.082) (**H**) across genotype. Hets: 83 cells, 21 animals and wts: 53 cells, 17 animals. (**I**) Example morphological reconstructions across genotype, cell bodies denoted with circles. Dendritic branch number (3-way ANOVA: genotype main effect: *F*_1, 40_ = 23.279, *P* < 0.001) (**J**), total dendritic branch length (3-way ANOVA: genotype main effect: *F*_1, 40_ = 7.002, *P* = 0.013) (**K**), and mean dendritic branch length (3-way ANOVA: genotype main effect: *F*_1, 40_ = 0.133, *P* = 0.718) (**L**) across genotype. **M** Scholl analysis across genotype (3-way ANOVA: genotype main effect: *F*_1, 31_ = 5.532, *P* = 0.025). Hets: 23 cells, 12 animals and wts: 17 cells, 9 animals. **N** Correlation between total dendritic branch length and input resistance dataset (Pearson correlation: *R*^2^ = 0.015, *P* = 0.600). Summary values depicted as mean ± SEM. *P < 0.05, **P < 0.01, ***P < 0.001 (3-way ANOVA between subject effect).

To assess input resistance, measurements were analysed from voltage clamp experiments (using identical conditions to the LTP experiments in Fig. 1) and in current clamp experiments. In both these separate and independent data sets *Dlg2*+*/−* hets had reduced input resistance (Fig. 4A–D). This increase in electrical leak in the *Dlg2*+*/−* hets is predicted to reduce cross-talk between synapses and their integration leading to a reduced spike output but it is also expected to reduce the spike output in response to somatic current injection. However, despite reduced input resistance in the *Dlg2*+*/−* hets, there was no effect of genotype on spike output to current injection (rheobase) (Fig. 4E, F). This could be explained by a depolarised resting membrane potential (Fig. 4G) and a trend towards hyperpolarised action potential spike threshold (Fig. 4H) in the *Dlg2*+*/−* hets indicating that smaller membrane potential depolarisations were required to initiate spikes. There was no effect of genotype on spike half-width, maximum spike slope, spike amplitude, or capacitance, and a slight decrease in latency to spike in the *Dlg2*+*/−* hets (Supplementary Fig. S16).

Reduced input resistance in the *Dlg2*+*/−* hets could be explained via two mechanisms: i) increased membrane area through greater dendritic branching and extent [42, 67] or ii) increased membrane conductance, most likely caused by increased potassium channel expression. To test the first mechanism, a subset of neurons from the intrinsic excitability experiments were filled with neurobiotin to allow *post hoc* morphological analysis. Analysis of these neurons revealed that het neurons were smaller than wt neurons (Fig. 4I) and had reduced dendritic branch number and total dendritic branch length but had similar mean dendritic branch lengths (Fig. 4J–L). Scholl analysis demonstrated that *Dlg2*+*/−* het neurons had reduced dendritic arborisation overall, with the most striking differences in the basal and proximal apical regions (Fig. 4M). Contrary to the predicted neuronal size – input resistance relationship, there was no correlation between total dendritic branch length and input resistance (Fig. 4N). Therefore, reduced neuronal arborisation in the *Dlg2*+*/−* hets cannot explain the observed reduced input resistance and instead increased potassium channel expression is the most likely explanation.

Computational modelling of synaptic integration in representative reconstructed pyramidal neurons also predicted increased potassium channel expression as the mechanism underlying reduced input resistance (Supplementary Fig. S19) and enabled exploration of the likely potassium channel subtypes mediating reduced synaptic integration. DLG2 interacts with potassium inward rectifier Kir2.3 [22] and Kir2.2 [23] as well as A-type Kv1.4 [24] channels which therefore represent potential candidates to underpin decreased input resistance and synaptic integration. The model suggested that A-type potassium channels are the most likely candidates upregulated in the *Dlg2*+*/−* hets to underly the dendritic integration deficits (Supplementary Fig. S19). However, any mechanism that reduces input resistance is predicted to facilitate dendritic integration in the *Dlg2*+/− hets.

### Rescue of synaptic integration and plasticity

**Fig. 5:**
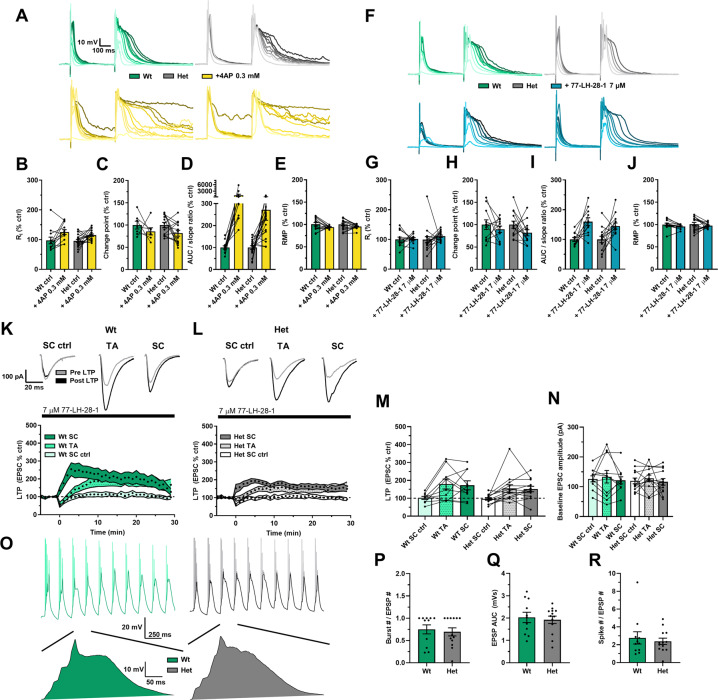
Potassium channel block and muscarinic M1 agonism lower dendritic integration thresholds and M1 agonism rescues associative LTP in the *Dlg2*+*/−* hets. **A** Example traces depicting a single EPSP followed by a compound EPSP at increasing stimulation intensities (light to dark) over consecutive recording sweeps before and after 4-aminopyridine 0.3 mM across genotype. Input resistance (3-way repeated-measures ANOVA: drug effect: *F*_1, 23_ = 8.608, *P* = 0.007, genotype x drug interaction: *F*_1, 23_ = 0.418, *P* = 0.524) (**B**), change point (3-way repeated-measures ANOVA: drug effect: *F*_1, 14_ = 8.422, *P* = 0.012, genotype x drug interaction: *F*_1, 14_ = 0.042, *P* = 0.841) (**C**), AUC/slope (3-way repeated-measures ANOVA: drug effect: *F*_1, 23_ = 3.988, *P* = 0.058, genotype x drug interaction: *F*_1, 23_ = 1.570, *P* = 0.223) (**D**), and resting membrane potential (RMP) (3-way repeated-measures ANOVA: drug effect: *F*_1, 24_ = 57.899, *P* < 0.001, genotype x drug interaction: *F*_1, 24_ = 0.230, *P* = 0.636) (**E**) as percent of control before and after the after 4-aminopyridine 0.3 mM across genotype. Hets: 19 cells, 10 animals and wts: 13 cells, 6 animals. **F** Example traces depicting a single EPSP followed by a compound EPSP at increasing stimulation intensities (light to dark) over consecutive recording sweeps before and after 77-LH-28-1 7 µM across genotype. Input resistance (3-way repeated-measures ANOVA: drug effect: *F*_1, 21_ = 4.12, *P* = 0.055, genotype x drug interaction: *F*_1, 21_ = 1.270, *P* = 0.273) (**G**), change point (3-way repeated-measures ANOVA: drug effect: *F*_1, 16_ = 6.879, *P* = 0.018, genotype x drug interaction: *F*_1, 16_ = 0.645, *P* = 0.434) (**H**), AUC/slope (3-way repeated-measures ANOVA: drug effect: *F*_1, 17_ = 38.074, *P* < 0.001, genotype x drug interaction: *F*_1, 17_ = 0.152, *P* = 0.701) (**I**), and resting membrane potential (RMP) (3-way repeated-measures ANOVA: drug effect: *F*_1, 21_ = 8.931, *P* = 0.007., genotype x drug interaction: *F*_1, 21_ = 1.412, *P* = 0.248) (**J**) as percent of control before and after the after 77-LH-28-1 7 µM. Hets: 17 cells, 7 animals and wts: 12 cells, 7 animals. aLTP over time in wts (**K**) and *Dlg2*+*/−* hets (**L**). Example traces pre- and post-induction are displayed for the wt and het groups above their corresponding plots of LTP over time. **M** aLTP at the 25–30 min mark post induction across genotype (3-way repeated-measures ANOVA: pathway effect: *F*_2, 32_ = 12.169, *P* < 0.001. Genotype main effect: *F*_1, 16_ = 0.176, *P* = 0.680. Genotype x pathway interaction: *F*_2, 32_ = 0.09, *P* = 0.914). **N** Baseline EPSC amplitude across genotype (3-way repeated-measures ANOVA: pathway effect: *F*_2, 32_ = 1.297, *P* = 0.287. Genotype main effect: *F*_1, 16_ < 0.001, *P* = 0.985. Genotype x pathway interaction: *F*_2, 32_ = 0.043, *P* = 0.958). **O** Example EPSP traces of LTP induction, with example EPSPs following *post hoc* spike truncation. Burst number (3-way ANOVA: genotype main effect: *F*_1, 16_ = 0.001, *P* = 0.974) (**P**), EPSP AUC (3-way ANOVA: genotype main effect: *F*_1, 16_ = 0.100, *P* = 0.755) (**Q**), and total spike number (3-way ANOVA: genotype main effect: *F*_1, 16_ = 0.029, *P* = 0.867) (**R**) across genotype during LTP induction. Hets: 13 cells, 7 animals and wts: 11 cells, 6 animals. Summary values depicted as mean ± SEM. *P < 0.05, **P < 0.01, ***P < 0.001 (3-way ANOVA between subject effect).

The aLTP, theta burst LTP, and dendritic integration results from Figs. 1 and 2 suggest that, given enough synaptic input, *Dlg2*+*/−* hets can express LTP despite their reduced input resistance. It follows that by increasing input resistance in the *Dlg2*+*/−* hets, dendritic integration and aLTP could be effectively rescued. Three separate methods to increase input resistance were tested for their effectiveness in rescuing dendritic integration. The first was the relatively broad-spectrum voltage-sensitive potassium channel blocker, 4-aminopyridine (4-AP) [68], the second was the selective Kv1.3, Kv1.4 blocker CP339818 [69] and the third was activation of muscarinic M1 receptors [54, 70]. 4-AP caused an increase in input resistance, a reduction in the supra-linearity change point, a trend toward increased maximum duration of the compound EPSP as a ratio to the corresponding slope of the single EPSP, and a repolarisation in resting membrane potential (Fig. 5A–E). The effects of 4-AP were not genotype-specific, as there were no drug x genotype interactions. These results support the computational modelling predictions that voltage-sensitive potassium channels attenuate dendritic integration and blocking them facilitates it. Since DLG2 interacts with Kv1.4, the selective blocker CP339818 was used to test whether the upregulation of these channels was responsible for reduced dendritic integration. However, CP339818 had no effect on input resistance or dendritic integration (Supplementary Fig. S20) indicating that upregulation of these specific A-type potassium channels does not underpin the reduction in dendritic integration in the *Dlg2*+*/−* hets but does not rule out a role for other A-type channels.

These results demonstrate, as predicted, that blocking a subset of potassium channels activated around the resting membrane potential facilitates dendritic integration. However, due to the considerable heterogeneity of potassium channels and their ability to compensate for one another coupled with limited availability of selective pharmacological tools, identifying and targeting the precise channels that cause reduced input resistance in the *Dlg2*+*/−* hets is challenging. An alternative approach, and one with greater therapeutic potential, is to rescue the input resistance reduction indirectly, for example by activation of cholinergic muscarinic M1 receptors that inhibit potassium channel function and increase dendritic excitability [42, 47, 57–61]. Support for this approach was found using the highly selective muscarinic M1 receptor allosteric partial agonist 77-LH-28-1 [71] which increased input resistance, reduced the change point, increased the maximum duration of the compound EPSP as a ratio to the corresponding slope of the single EPSP, and depolarised the resting membrane potential (Fig. 5F–J). However, there were no significant drug x genotype interactions. Similar results were found for the broad-spectrum non-hydrolysable acetylcholine analogue carbachol (Supplementary Fig. S21).

These results suggest that pharmacological enhancement of dendritic excitability and integration may be sufficient to rescue aLTP in the *Dlg2*+*/−* hets. Therefore, the aLTP experiment was repeated in the presence of 77-LH-28-1. This rescued aLTP in the *Dlg2*+*/−* hets with robust aLTP in SC and TA pathways (Fig. 5K–M). In addition, unlike in the absence of 77-LH-28-1, there was no effect of genotype and no pathway x genotype interaction (Fig. 5M), indicating 77-LH-28-1 selectively rescues aLTP in the *Dlg2*+*/−* hets. Importantly, baseline EPSC amplitude did not differ among pathways and across genotype (Fig. 5N), indicating that the amount of synaptic input received was similar in all conditions. Analysis of the aLTP induction phase revealed that 77-LH-28-1 rescued synaptic summation and the resulting action potential spiking (Fig. 5O–R) as well as plateau potential generation (Supplementary Fig. S22), with the genotypic differences for number of bursts, EPSP summation, and spike number disappearing. Taken together, Fig. 5 shows 77-LH-28-1 reduced the threshold for dendritic integration in both wts and *Dlg2*+*/−* hets but selectively facilitated aLTP in the *Dlg2*+*/−* hets indicating induction of synaptic plasticity in the *Dlg2*+*/−* hets is more sensitive to increased dendritic excitability and synaptic integration.

## Discussion

NMDAR currents are increased in the *Dlg2*+*/−* heterozygous rat model. Additionally, dendritic arborisation is reduced. These observations would be expected to combine to enhance neuronal excitability, dendritic integration and synaptic plasticity. Instead, the effects are entirely offset, and indeed reversed, by a concomitant reduction in input resistance caused by an increase in potassium channel expression, potentially A-type potassium channels. This increase in electrical leak is the dominant effect, resulting in a final phenotype where dendritic integration and aLTP are impaired. Crucially, dendritic integration can be rescued by potassium channel block or activation of muscarinic M1 receptors, the latter of which can also rescue synaptic plasticity. These phenotypes are potentially particularly relevant since the *Dlg2*+*/−* rat model relates to human single copy genetic variants.

The direct interaction between DLG2 and GluN2b NMDAR subunits suggest the most important effects of DLG2 perturbations are on NMDAR function – synaptic integration and plasticity. However, previous studies on *Dlg2−/−* full knockout models have either reported no changes in the AMPA/NMDA ratio or a reduction in the AMPA/NMDA ratio due to reduction in AMPAR function [32–34, 48, 49], Here, AMPAR function was unchanged and instead we found an unexpected increase in NMDAR currents, likely caused by increased synaptic expression selectively at Schaffer collateral synapses. There is no evidence that DLG2 is differentially expressed at Schaffer collateral vs temporoammonic synapses in CA1 so the mechanism for this selective enhancement of NMDAR expression is unknown. On its own, enhanced NMDAR currents predict enhanced aLTP, but we found the converse with aLTP impairment. This is similar to previous reports in *Dlg2−/−* mice. In homozygous *Dlg2−/−* mice CA1 LTP was normal in response to strong 100 Hz induction protocol but reduced in response to TBS given to just the SC pathway [33]. In our study using heterozygous *Dlg2*+*/−* rats, TBS-induced LTP pairing postsynaptic stimulation with SC input was normal and an LTP deficit only became apparent in the *Dlg2*+*/−* model when neurons were required to integrate converging inputs suggesting a nuanced and potentially behaviourally relevant phenotype in the clinically relevant *Dlg2*+*/−* model. Furthermore, synaptic integration and the initiation of non-linear dendritic events are key determinants of feature detection and selectivity in neuronal networks [41, 72, 73] and a deficit in detecting events and giving appropriate salience are important features of many psychiatric disorders [74].

The dichotomy between enhanced NMDA currents and reduced NMDAR function in *Dlg2*+*/−* rats during aLTP highlights the dominant role played by changes to intrinsic neuronal excitability; in this instance reduced input resistance caused by increases in potassium channel function. Interestingly, in a *Dlg2−/−* full knockout model no changes in input resistance were reported [75] highlighting again the importance of using clinically relevant models. In our *Dlg2*+*/−* model this increase in potassium channel function does not appear to be caused by a direct interaction with DLG2 but instead as a homoeostatic regulatory mechanism perhaps to compensate for increased synaptic currents. A similar compensatory mechanism is found in other models of psychiatric disorders such as *Fmr1-/y* mice where changes in intrinsic neuronal excitability dominate the resulting perturbations in network processing including dendritic integration and synaptic plasticity [63, 76–78]. This raises the intriguing possibility that genetic disruptions to synaptic function may generally cause homoeostatic compensations in intrinsic neuronal excitability that dominate neuronal function and present a common biological phenotype across multiple psychiatric disorders [79].

We have demonstrated in this study that the compensatory mechanisms affecting neuronal excitability can be ameliorated pharmacologically with the administration of selective agonists such as 77-LH-28-1 rescuing impairments in synaptic integration and plasticity, a proof of principle that may be applicable to other psychiatric disorder risk variants. For example, an increase in input resistance due to the administration of 77-LH-28-1 could facilitate spike backpropagation, potentially rescuing the plasticity impairment and network dysfunctions reported in the *Cacna1c*+*/−* and 22q11 deletion syndrome models of genetic vulnerability to schizophrenia [80, 78], Highly selective muscarinic M1 receptor agonists have efficacy clinically with negligible side effects [81–84] making them attractive pharmaceutical tools. It remains to be seen whether behavioural impairments in DLG2 models can be rescued using similar pharmacological strategies.

### Supplementary information

supplemental 1

supplement 2

## References

[1] Kirov G, Pocklington AJ, Holmans P, Ivanov D, Ikeda M, Ruderfer D (2012). De novo CNV analysis implicates specific abnormalities of postsynaptic signalling complexes in the pathogenesis of schizophrenia. Mol Psychiatry.

[2] Marshall CR, Howrigan DP, Merico D, Thiruvahindrapuram B, Wu W, Greer DS (2017). Contribution of copy number variants to schizophrenia from a genome-wide study of 41,321 subjects. Nat Genet.

[3] Noor A, Lionel AC, Cohen-Woods S, Moghimi N, Rucker J, Fennell A (2014). Copy number variant study of bipolar disorder in Canadian and UK populations implicates synaptic genes. Am J Med Genet Part B Neuropsychiatr Genet.

[4] Georgieva L, Rees E, Moran JL, Chambert KD, Milanova V, Craddock N (2014). De novo CNVs in bipolar affective disorder and schizophrenia. Hum Mol Genet.

[5] Cusco I, Medrano A, Gener B, Vilardell M, Gallastegui F, Villa O (2009). Autism-specific copy number variants further implicate the phosphatidylinositol signaling pathway and the glutamatergic synapse in the etiology of the disorder. Hum Mol Genet.

[6] Egger G, Roetzer KM, Noor A, Lionel AC, Mahmood H, Schwarzbraun T (2014). Identification of risk genes for autism spectrum disorder through copy number variation analysis in Austrian families. Neurogenetics.

[7] Ruzzo EK, Pérez-Cano L, Jung J-Y, Wang L-k, Kashef-Haghighi D, Hartl C (2019). Inherited and de novo genetic risk for autism impacts shared networks. Cell.

[8] Alemany S, Ribasés M, Vilor-Tejedor N, Bustamante M, Sánchez-Mora C, Bosch R (2015). New suggestive genetic loci and biological pathways for attention function in adult attention-deficit/hyperactivity disorder. Am J Med Genet Part B Neuropsychiatr Genet.

[9] Reggiani C, Coppens S, Sekhara T, Dimov I, Pichon B, Lufin N (2017). Novel promoters and coding first exons in DLG2 linked to developmental disorders and intellectual disability. Genome Med.

[10] Vulto-van Silfhout AT, Hehir-Kwa JY, van Bon BWM, Schuurs-Hoeijmakers JHM, Meader S, Hellebrekers CJM (2013). Clinical significance of de novo and inherited copy-number variation. Hum Mutat.

[11] Ferrari R, Kia DA, Tomkins JE, Hardy J, Wood NW, Lovering RC (2018). Stratification of candidate genes for Parkinson’s disease using weighted protein-protein interaction network analysis. BMC Genomics.

[12] Wu H-C, Chen C-M, Chen Y-C, Fung H-C, Chang K-H, Wu Y-R (2018). DLG2, but not TMEM229B, GPNMB, and ITGA8 polymorphism, is associated with Parkinson’s disease in a Taiwanese population. Neurobiol Aging.

[13] Fromer M, Pocklington AJ, Kavanagh DH, Williams HJ, Dwyer S, Gormley P (2014). De novo mutations in schizophrenia implicate synaptic networks. Nature..

[14] Purcell SM, Moran JL, Fromer M, Ruderfer D, Solovieff N, Roussos P (2014). A polygenic burden of rare disruptive mutations in schizophrenia. Nature..

[15] Gonzalez-Mantilla AJ, Moreno-De-Luca A, Ledbetter DH, Martin CL (2016). A cross-disorder method to identify novel candidate genes for developmental brain disorders. JAMA Psychiatry.

[16] Sanders SJ, Murtha MT, Gupta AR, Murdoch JD, Raubeson MJ, Willsey AJ (2012). De novo mutations revealed by whole-exome sequencing are strongly associated with autism. Nature..

[17] Irie M (1997). Binding of neuroligins to PSD-95. Science.

[18] Niethammer M, Kim E, Sheng M (1996). Interaction between the C terminus of NMDA receptor subunits and multiple members of the PSD-95 family of membrane-associated guanylate kinases. J Neurosci.

[19] Chen B-S, Gray John A, Sanz-Clemente A, Wei Z, Thomas Eleanor V, Nicoll Roger A (2012). SAP102 mediates synaptic clearance of NMDA Receptors. Cell Rep.

[20] Frank RAW, Komiyama NH, Ryan TJ, Zhu F, O’Dell TJ, Grant SGN (2016). NMDA receptors are selectively partitioned into complexes and supercomplexes during synapse maturation. Nat Commun.

[21] Dakoji S, Tomita S, Karimzadegan S, Nicoll RA, Bredt DS (2003). Interaction of transmembrane AMPA receptor regulatory proteins with multiple membrane associated guanylate kinases. Neuropharmacology..

[22] Inanobe A, Fujita A, Ito M, Tomoike H, Inageda K, Kurachi Y (2002). Inward rectifier K+ channel Kir2.3 is localized at the postsynaptic membrane of excitatory synapses. Am J Physiol Cell Physiol.

[23] Leonoudakis D, Conti LR, Anderson S, Radeke CM, McGuire LMM, Adams ME (2004). Protein trafficking and anchoring complexes revealed by proteomic analysis of inward rectifier potassium channel (Kir2.x)-associated proteins. J Biol Chem.

[24] Kim E, Cho K-O, Rothschild A, Sheng M (1996). Heteromultimerization and NMDA receptor-clustering activity of Chapsyn-110, a member of the PSD-95 family of proteins. Neuron..

[25] Sanders SS, Hernandez LM, Soh H, Karnam S, Walikonis RS, Tzingounis AV (2020). The palmitoyl acyltransferase ZDHHC14 controls Kv1-family potassium channel clustering at the axon initial segment. eLife..

[26] Nada S, Shima T, Yanai H, Husi H, Grant SGN, Okada M (2003). Identification of PSD-93 as a substrate for the Src family tyrosine kinase Fyn. J Biol Chem.

[27] Sato Y, Tao YX, Su Q, Johns RA (2008). Post-synaptic density-93 mediates tyrosine-phosphorylation of the N-methyl-d-aspartate receptors. Neuroscience.

[28] Guo M-L, Xue B, Jin D-Z, Mao L-M, Wang JQ (2012). Interactions and phosphorylation of postsynaptic density 93 (PSD-93) by extracellular signal-regulated kinase (ERK). Brain Res.

[29] Kim E, Naisbitt S, Hsueh Y-P, Rao A, Rothschild A, Craig AM (1997). GKAP, a novel synaptic protein that interacts with the guanylate kinase-like domain of the PSD-95/SAP90 family of channel clustering molecules. J Cell Biol.

[30] Brenman JE, Topinka JR, Cooper EC, McGee AW, Rosen J, Milroy T (1998). Localization of postsynaptic density-93 to dendritic microtubules and interaction with microtubule-associated protein 1A. J Neurosci.

[31] Ogawa Y, Horresh I, Trimmer JS, Bredt DS, Peles E, Rasband MN (2008). Postsynaptic density-93 clusters Kv1 channels at axon initial segments independently of Caspr2. J Neurosci.

[32] Elias GM, Funke L, Stein V, Grant SG, Bredt DS, Nicoll RA (2006). Synapse-specific and developmentally regulated targeting of AMPA receptors by a family of MAGUK scaffolding proteins. Neuron..

[33] Carlisle HJ, Fink AE, Grant SGN, O’Dell TJ (2008). Opposing effects of PSD-93 and PSD-95 on long-term potentiation and spike timing-dependent plasticity: membrane-associated guanylate kinases and synaptic plasticity. J Physiol.

[34] Krüger JM, Favaro PD, Liu M, Kitlińska A, Huang X, Raabe M (2013). Differential roles of postsynaptic density-93 isoforms in regulating synaptic transmission. J Neurosci.

[35] Yoo T, Kim S-G, Yang SH, Kim H, Kim E, Kim SY (2020). A DLG2 deficiency in mice leads to reduced sociability and increased repetitive behavior accompanied by aberrant synaptic transmission in the dorsal striatum. Mol Autism.

[36] Nithianantharajah J, Komiyama NH, McKechanie A, Johnstone M, Blackwood DH, Clair DS (2013). Synaptic scaffold evolution generated components of vertebrate cognitive complexity. Nat Neurosci.

[37] Winkler D, Daher F, Wüstefeld L, Hammerschmidt K, Poggi G, Seelbach A (2018). Hypersocial behavior and biological redundancy in mice with reduced expression of PSD95 or PSD93. Behavioural Brain Res.

[38] Chen X, Fukata Y, Fukata M, Nicoll RA. MAGUKs are essential, but redundant, in long-term potentiation. Proc Natl Acad Sci USA. 2021;118:e2107585118. 10.1073/pnas.2107585118 PMC8285982 34244435

[39] Waldron S, Pass R, Griesius S, Mellor JR, Robinson ESJ, Thomas KL, et al. Behavioural and molecular characterisation of the Dlg2 haploinsufficiency rat model of genetic risk for psychiatric disorder. bioRxiv. 2021;2021.08.02.454736. 10.1111/gbb.12797 PMC9393932 35075790

[40] Cornford JH, Mercier MS, Leite M, Magloire V, Häusser M, Kullmann DM (2019). Dendritic NMDA receptors in parvalbumin neurons enable strong and stable neuronal assemblies. eLife..

[41] Bittner KC, Grienberger C, Vaidya SP, Milstein AD, Macklin JJ, Suh J (2015). Conjunctive input processing drives feature selectivity in hippocampal CA1 neurons. Nat Neurosci.

[42] Poleg-Polsky A (2015). Effects of neural morphology and input distribution on synaptic processing by global and focal NMDA-spikes. PLOS One.

[43] Takahashi H, Magee JC (2009). Pathway interactions and synaptic plasticity in the dendritic tuft regions of CA1 pyramidal neurons. Neuron..

[44] Jarsky T, Roxin A, Kath WL, Spruston N (2005). Conditional dendritic spike propagation following distal synaptic activation of hippocampal CA1 pyramidal neurons. Nat Neurosci.

[45] Larkum ME, Zhu JJ, Sakmann B (1999). A new cellular mechanism for coupling inputs arriving at different cortical layers. Nature..

[46] Magee JC (1997). A synaptically controlled, associative signal for Hebbian plasticity in hippocampal neurons. Science..

[47] Stuart GJ, Spruston N (2015). Dendritic integration: 60 years of progress. Nat Neurosci.

[48] Favaro PD, Huang X, Hosang L, Stodieck S, Cui L, Liu YZ (2018). An opposing function of paralogs in balancing developmental synapse maturation. PLoS Biol.

[49] Tao Y-X, Rumbaugh G, Wang G-D, Petralia RS, Zhao C, Kauer FW (2003). Impaired NMDA receptor-mediated postsynaptic function and blunted NMDA receptor-dependent persistent pain in mice lacking postsynaptic density-93 protein. J Neurosci.

[50] Stuart G, Spruston N (1998). Determinants of voltage attenuation in neocortical pyramidal neuron dendrites. J Neurosci.

[51] Magee JC (1998). Dendritic hyperpolarization-activated currents modify the integrative properties of hippocampal CA1 pyramidal neurons. J Neurosci.

[52] Lorincz A, Notomi T, Tamas G, Shigemoto R, Nusser Z (2002). Polarized and compartment-dependent distribution of HCN1 in pyramidal cell dendrites. Nat Neurosci.

[53] Harnett MT, Magee JC, Williams SR (2015). Distribution and function of HCN channels in the apical dendritic tuft of neocortical pyramidal neurons. J Neurosci.

[54] Buchanan KA, Petrovic MM, Chamberlain SEL, Marrion NV, Mellor JR (2010). Facilitation of long-term potentiation by muscarinic M1 receptors is mediated by inhibition of SK channels. Neuron..

[55] Tigaret CM, Olivo V, Sadowski JHLP, Ashby MC, Mellor JR (2016). Coordinated activation of distinct Ca2+ sources and metabotropic glutamate receptors encodes Hebbian synaptic plasticity. Nat Commun..

[56] Ngo-Anh TJ, Bloodgood BL, Lin M, Sabatini BL, Maylie J, Adelman JP (2005). SK channels and NMDA receptors form a Ca2+-mediated feedback loop in dendritic spines. Nat Neurosci.

[57] Harnett Mark T, Xu N-L, Magee Jeffrey C, Williams Stephen R (2013). Potassium channels control the interaction between active dendritic integration compartments in Layer 5 cortical pyramidal neurons. Neuron..

[58] Biró ÁA, Brémaud A, Falck J, Ruiz AJ (2018). A-type K+ channels impede supralinear summation of clustered glutamatergic inputs in layer 3 neocortical pyramidal neurons. Neuropharmacology..

[59] Losonczy A, Magee JC (2006). Integrative properties of radial oblique dendrites in hippocampal CA1 pyramidal neurons. Neuron..

[60] Liu Y, Cui L, Schwarz MK, Dong Y, Schlüter OM (2017). Adrenergic gate release for spike timing-dependent synaptic potentiation. Neuron..

[61] Makara Judit K, Magee Jeffrey C (2013). Variable dendritic integration in hippocampal CA3 pyramidal neurons. Neuron..

[62] Dougherty KA, Nicholson DA, Diaz L, Buss EW, Neuman KM, Chetkovich DM (2013). Differential expression of HCN subunits alters voltage-dependent gating of h-channels in CA1 pyramidal neurons from dorsal and ventral hippocampus. J Neurophysiol.

[63] Booker SA, Domanski APF, Dando OR, Jackson AD, Isaac JTR, Hardingham GE (2019). Altered dendritic spine function and integration in a mouse model of fragile X syndrome. Nat Commun.

[64] Fan Y, Fricker D, Brager DH, Chen X, Lu H-C, Chitwood RA (2005). Activity-dependent decrease of excitability in rat hippocampal neurons through increases in Ih. Nat Neurosci.

[65] Brager DH, Lewis AS, Chetkovich DM, Johnston D (2013). Short- and long-term plasticity in CA1 neurons from mice lacking h-channel auxiliary subunit TRIP8b. J Neurophysiol.

[66] Narayanan R, Johnston D (2007). Long-term potentiation in rat hippocampal neurons is accompanied by spatially widespread changes in intrinsic oscillatory dynamics and excitability. Neuron..

[67] Mihaljević B, Larrañaga P, Bielza C (2021). Comparing the electrophysiology and morphology of human and mouse layer 2/3 pyramidal neurons with bayesian networks. Front Neuroinformatics.

[68] Armstrong JF, Faccenda E, Harding SD, Pawson AJ, Southan C, Sharman JL (2020). The IUPHAR/BPS guide to PHARMACOLOGY in 2020: extending immunopharmacology content and introducing the IUPHAR/MMV Guide to MALARIA PHARMACOLOGY. Nucleic Acids Res.

[69] Nguyen A, Kath J, Hanson D, Biggers M, Canniff P, Donovan C (1996). Novel nonpeptide agents potently block the C-type inactivated conformation of Kv1.3 and suppress T cell activation. Mol Pharmacol.

[70] Dennis SH, Pasqui F, Colvin EM, Sanger H, Mogg AJ, Felder CC (2016). Activation of muscarinic M1 acetylcholine receptors induces long-term potentiation in the hippocampus. Cereb Cortex.

[71] Langmead CJ, Austin NE, Branch CL, Brown JT, Buchanan KA, Davies CH (2008). Characterization of a CNS penetrant, selective M1 muscarinic receptor agonist, 77-LH-28-1: 77-LH-28-1, a selective M1 mAChR agonist. Br J Pharmacol.

[72] Takahashi N, Oertner TG, Hegemann P, Larkum ME (2016). Active cortical dendrites modulate perception. Science.

[73] Takahashi N, Ebner C, Sigl-Glockner J, Moberg S, Nierwetberg S, Larkum ME (2020). Active dendritic currents gate descending cortical outputs in perception. Nat Neurosci.

[74] American Psychiatric Association. Diagnostic and statistical manual of mental disorders. 5th ed. Washington, DC; American Psychiatric Association: 2013.

[75] Sanders B, D’Andrea D, Collins MO, Rees E, Steward TGJ, Zhu Y, et al. DLG2 knockout reveals neurogenic transcriptional programs underlying neuropsychiatric disorders and cognition. bioRxiv. 2020;2020.01.10.898676.

[76] Domanski APF, Booker SA, Wyllie DJA, Isaac JTR, Kind PC (2019). Cellular and synaptic phenotypes lead to disrupted information processing in Fmr1-KO mouse layer 4 barrel cortex. Nat Commun..

[77] Mi Z, Yang J, He Q, Zhang X, Xiao Y, Shu Y (2019). Alterations of electrophysiological properties and ion channel expression in prefrontal cortex of a mouse model of schizophrenia. Front Cell Neurosci.

[78] Marissal T, Salazar RF, Bertollini C, Mutel S, De Roo M, Rodriguez I (2018). Restoring wild-type-like CA1 network dynamics and behavior during adulthood in a mouse model of schizophrenia. Nat Neurosci.

[79] Mizusaki BEP, O’Donnell C. Neural circuit function redundancy in brain disorders. Curr Opin Neurobiol. 2021;70:74–80. 10.1016/j.conb.2021.07.008 PMC8694099 34416675

[80] Tigaret CM, Lin T-CE, Morrell ER, Sykes L, Moon AL, O’Donovan MC, et al. Neurotrophin receptor activation rescues cognitive and synaptic abnormalities caused by hemizygosity of the psychiatric risk gene Cacna1c. Mol Psychiatry. 2021;26:1748–1760. 10.1038/s41380-020-01001-0 PMC8440217 33597718

[81] Nathan PJ, Watson J, Lund J, Davies CH, Peters G, Dodds CM (2013). The potent M1 receptor allosteric agonist GSK1034702 improves episodic memory in humans in the nicotine abstinence model of cognitive dysfunction. Int J Neuropsychopharmacol.

[82] Bakker C, van der Aart J, Labots G, Liptrot J, Cross DM, Klaassen ES, et al. Safety and pharmacokinetics of HTL0018318, a novel M1 receptor agonist, given in combination with donepezil at steady state: a randomized trial in healthy elderly subjects. Drugs R D. 2021;21:295–304. 10.1007/s40268-021-00352-5 PMC8363684 34164794

[83] Bakker C, Tasker T, Liptrot J, Hart EP, Klaassen ES, Doll RJ (2021). Safety, pharmacokinetics and exploratory pro-cognitive effects of HTL0018318, a selective M1 receptor agonist, in healthy younger adult and elderly subjects: a multiple ascending dose study. Alzheimers Res Ther.

[84] Shekhar A, Potter WZ, Lightfoot J, Lienemann J, Dubé S, Mallinckrodt C (2008). Selective muscarinic receptor agonist xanomeline as a novel treatment approach for schizophrenia. Am J Psychiatry.

