# Correction: Reduced expression of the psychiatric risk gene DLG2 (PSD93) impairs hippocampal synaptic integration and plasticity

**DOI:** 10.1038/s41386-024-01801-w

**Published:** 2024-01-15

**Authors:** Simonas Griesius, Cian O’Donnell, Sophie Waldron, Kerrie L. Thomas, Dominic M. Dwyer, Lawrence S. Wilkinson, Jeremy Hall, Emma S. J. Robinson, Jack R. Mellor

**Affiliations:** 1 Centre for Synaptic Plasticity, School of Physiology, Pharmacology and Neuroscience, University of Bristol, University Walk, Bristol, BS8 1TD UK; 2 Computational Neuroscience Unit, School of Computer Science, Electrical and Electronic Engineering, and Engineering Mathematics, University of Bristol, Bristol, BS8 1UB UK; 3 Neuroscience and Mental Health Research Institute, Cardiff, CF24 4HQ UK; 4 School of Psychology, Cardiff, CF24 4HQ UK; 5 School of Medicine, Cardiff, CF24 4HQ UK; 6 MRC Centre for Neuropsychiatric Genetics and Genomics, Cardiff, CF24 4HQ UK

**Keywords:** Cellular neuroscience, Long-term potentiation, Schizophrenia, Risk factors

Correction to: *Neuropsychopharmacology* 10.1038/s41386-022-01277-6, published online 03 February 2022

The Wellcome funder grant number 101029/Z/13/Z was missing from the ‘Funding’ section in the original article. The original article has been corrected.

