# Supplementary material for: Reduced expression of the psychiatric risk gene DLG2 (PSD93) impairs hippocampal synaptic integration and plasticity
Source: Neuropsychopharmacology. Author manuscript; Available in PMC 2022 May 22. (PMC9117295; doi:10.1038/s41386-022-01277-6)
Supplement: supplemental 1 [file EMS140795-supplement-supplemental_1.pdf]

Supplementary information – Supplement 1

Reduced expression of the psychiatric risk gene DLG2 (PSD93) impairs hippocampal synaptic integration and plasticity

Simonas Griesius[1], Cian O'Donnell[2], Sophie Waldron[3,6], Kerrie L. Thomas[3,5], Dominic M. Dwyer[3,6], Lawrence S. Wilkinson[3,4,6], Jeremy Hall[3,4,5], Emma S. J. Robinson[1], Jack R. Mellor[1*]

[1] Centre for Synaptic Plasticity, School of Physiology, Pharmacology and Neuroscience, University of Bristol, University Walk, Bristol BS8 1TD, UK

[2] Computational Neuroscience Unit, School of Computer Science, Electrical and Electronic Engineering, and Engineering Mathematics, University of Bristol, Bristol BS8 1UB, UK

[3] Neuroscience and Mental Health Research Institute, [4] MRC Centre for Neuropsychiatric Genetics and Genomics, Schools of [5] Medicine and [6] Psychology, Cardiff CF24 4HQ, UK

* Corresponding author: Jack.Mellor@Bristol.ac.uk, +44 117 331 1944

Contents

CRISPR-Cas9 generation of Dlg2+/- heterozygous rat model and quality control measures (including Figures S1, S2 and Table S1).

Methods: brain slice preparation, electrophysiology, protein quantification and computational modelling

Table S2 peak channel conductances used in computational modelling simulations

Table S3 model parameters used in computational modelling simulations

Figure S3 DLG2 expression is reduced in Dlg2+/- hets, with no change in DLG4 or GluN1 expression

Figure S4 Example pathway independence check from the aLTP experiments

Figure S5 Spikes and AUC are positively correlated with aLTP in the SC but not the TA pathway

Figure S6 Increased baseline EPSC amplitude produces aLTP in both genotypes

Figure S7 Increased baseline EPSC amplitude facilitates putative plateau potential generation and aLTP

Figure S8 Attenuated aLTP in the dorsal hippocampus

Figure S9 Attenuated theta burst LTP in the ventral aspect of the hippocampus

Figure S10 mEPSC are blocked by NBQX

Figure S11 Paired pulse facilitation (AMPA/NMDA ratio experiment)

Figure S12 Unaltered HCN and SK channel function in the Dlg2+/- hets.

Figure S13 No effect of genotype on impedance and resonant frequency

Figure S14 Reduced impedance in the ventral aspect of the hippocampus

Figure S15 Increased impedance in neurons from female rats

Figure S16 The effect of genotype on additional intrinsic properties

Figure S17 The effect of dorsal-ventral aspect of the hippocampus on intrinsic properties

Figure S18 The effect of sex on intrinsic properties

Figure S19 With equal ionic conductance, simulated het neurons have higher input resistance. Ka channels reduce input resistance and inhibit dendritic integration

Figure S20 Blocking Kv1.3 and Kv1.4 channels selectively does not affect dendritic integration in the Dlg2+/- hets
Figure S21 Cholinergic low-dose agonism lowers dendritic integration thresholds in the Dlg2+/- hets
Figure S22 Muscarinic M1 agonism facilitates plateau potential generation in aLTP induction
References

The *Dlg2*+/- heterozygous rat model was created by Horizon Discovery (St Louis, Missouri, USA). Proprietary bioinformatics software (Horizon Discovery, St. Louis, USA) was used to design a short guide RNA (sgRNA - CCAGGGTCATCTCCAATGTGagg) targeting a Protospacer Adjacent Motif (PAM) sequence within exon 5 of the rat Dlg2 gene on chromosome 1. As shown in Fig S1 the targeting was predicted to result in a 7bp deletion (782933-782939 in the genomic sequence) and consequent downstream frame shift in exon 6 leading to a premature stop codon.

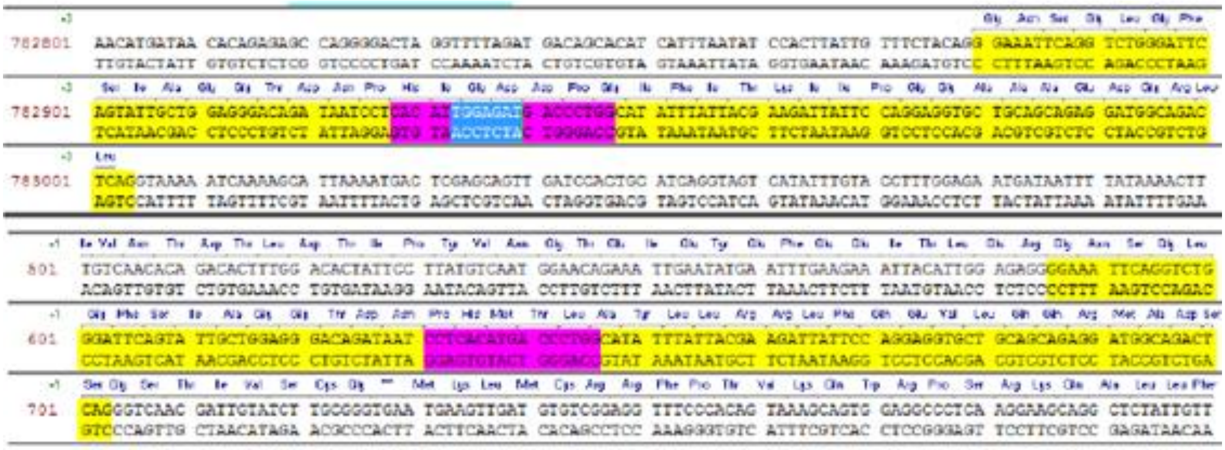

Figure S1 Targeting of exon 5 with deletion shown in blue (top panel) and outcome after non-homologous end joining in purple (lower panel)

Prior to the generation of the founder rats an initial in-vitro confirmation of the efficiency of the sgRNA-Cas9 was demonstrated by nucleofecting the sgRNA-Cas9 into rat C6 glial cells. Genomic DNA (gDNA) PCR products were subsequently generated from nucleofected C6 cells using primers flanking the sgRNA site. gDNA PCR products were screened for non-homologous end joining activity and deletion mutations using the SURVEYOR Cel-1 Mutation Detection Assay (Integrated DNA Technologies). Founder rats were then produced as follows; embryo donor female Long Evans rats were super-ovulated with pregnant mare serum (PMS) and given human chorionic gonadotrophin (HCG) 48 hrs post PMS administration. Females

were immediately mated to stud males after HCG administration. Embryo donor females were euthanized 18-24 hrs after mating and their one-cell fertilized embryos were isolated by harvesting the reproductive tract and rupturing the ampulae. Harvested embryos were put in culture media in a CO2 incubator until ready for microinjection. One-cell stage embryos were microinjected with the validated sgRNA-Cas9 and then implanted into synchronized pseudo-pregnant Long Evans recipient females.

The success of the targeting strategy was confirmed in founder rats using sequencing of gDNA PCR products derived from P14 tissue biopsies. Fig S2 illustrates genomic sequencing of a Dlg2 7bp out of frame heterozygous deletion founder. Manual reading of each double peak in the sequencing chromatograph (ABI Sequence Scanner) shows that upstream of the deletion, sequences from the wild-type and modified allele are identical. At the site of the deletion i) the sequence read becomes mixed and ii) the sequence of the secondary peaks from the modified allele align with wild-type sequence, except they occur 7bp further upstream revealing the size and position of the modified allele. As detailed in Fig S3-4, the effects of the predicted downstream premature stop codon in exon 6 was confirmed by Dlg2 expression analysis which showed the anticipated c.50% reduction in Dlg2 expression in the heterozygotes.

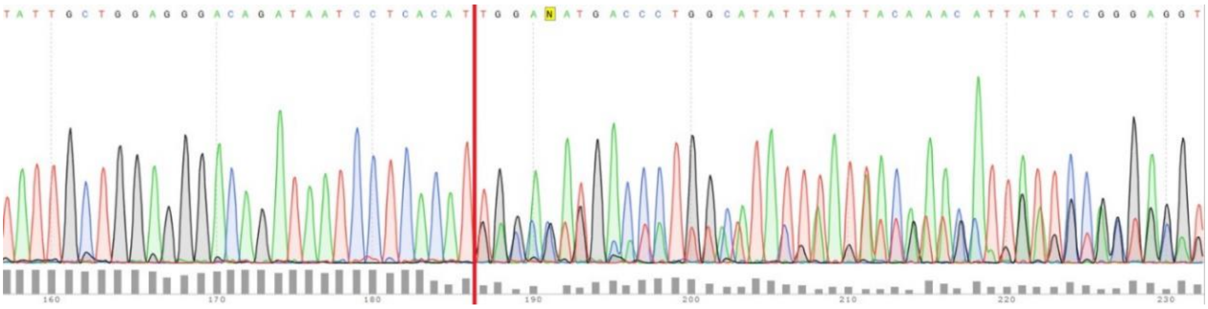

Figure S2 Sequencing of gDNA PCR products from wild-type and modified allele. The deletion site is marked by a red line, prior to which single peaks are seen and after which the peaks in the modified allele show the effects of the deletion moving them 7bp upstream. Data provided by Horizon Discovery and analysed using Snapgene Viewer 3.3.2.

Possible off-target hits were assessed by generating a list of top 10 potential off-target (OT) sites, based on the sgRNA sequence used (CCAGGGTCATCTCCAATGTGagg) and ranked using the MIT website http://crispr.mit.edu/. The top 10 OT sites were computed by taking into account the following i) total number of mismatches, ii) mismatch absolute position (to accommodate for the relatively high disturbance of mismatches falling close to the PAM site) and iii) mean pairwise distance between mismatches to account for the steric effect of closely neighbouring mismatches in disrupting sgRNA-DNA interaction. Corresponding PCR primer pairs were designed to flank the top 10 potential off target sites. Using extracted gDNA from the founder animal and wild-type controls, gDNA products were generated to flank each potential OT site (~300-500bp amplicon) and run on the SURVEYOR assay. Confirming the specificity of the CRISPR-Cas9 targeting to exon 5 of the Dlg2 gene none of the 10 OT sites tested revealed NHEJ activity (Table S1).

Founder rats were mated with wild types at Horizon Discovery and generated F2 progeny containing the mutation, thus confirming germ-line transmission. A total of five male positives were exported to Charles River, Lyon, France for re-derivation by embryo transfer. The resulting specific pathogen free (SPF) progeny were sent to Charles River, Margate, UK for routine breeding and maintenance of the lines. The standard breeding protocol was a heterozygous x wild-type cross giving rise to 1:1 average *Dlg2+/-* /wild-type progeny allowing full use of the litter and the generation of littermate controls. The *Dlg2+/-* rat model is viable and Charles River has reported no adverse effects on breeding performance, development, general health and in addition, no deviation from the expected Medelian 1:1 ratio of Dlg2+/- to wild-types and no skewing of the sex ratios. For the current experiments a cohort of breeders was transported to University of Bristol Animal Service Unit and the experimental animals used in the work were generated using heterozygous x wild-type cross giving rise to 1:1 average *Dlg2+/-* /wild-type littermate control progeny.

Table S1: List of top 10 most likely off-target sites (OT) when employing the sgRNA used to make the 7bp deletion in exon 5 of the Dlg2 gene generated using the in-silico MIT online tool (http://crispr.mit.edu/). As shown, gDNA PCR products amplified from the 10 possible off-target sites were all negative (i.e. showed no evidence of deletion or any other genomic change) when screened by the SURVEYOR assay. Data provided by Horizon Discovery.

Injected sgRNA sequence**:** CCAGGGTCATCTCCAATGTGagg

OT Site 1 CCAGGGTCATCTCCAATGTTAAG chr19: 34563666-34563688 screened by SURVEYOR aggttagtccgtgcatggtg gcttgcctacagccgtattt - <u>negative</u>

OT Site 2 CTTGAGTCATCTCCAATGTGTGG chr11: 75326973-75326995 screened by SURVEYOR tgtcagtgctgccttttgtc tcctttgtgtggtgtggttc - <u>negative</u>

OT Site 3 CCAGGGTGATCTCCAATCTGAGG chr14: 84150501-84150523 screened by SURVEYOR ggtaactggcctttgggttt tctgatttggggcttaggtg - <u>negative</u>

OT Site 4 TTATTGTCATCTCCAATGTGTAG chr16: 22863405-22863427 screened by SURVEYOR tacccacttttcacccaagc ttgccccttcagagaagac - <u>negative</u>

OT Site 5 CTATGGTCATCTCCAATGGGTAG chr20: 16439248-16439270 screened by SURVEYOR aaaccggttatgtcctgtgc ggaggaagatggagggaaac - <u>negative</u>

OT Site 6 ACAAGCTTATCTCCAATGTGTAG chr6: 46008366-46008388 screened by SURVEYOR tgcctaggaaactggcaact tgtgtcacttggatggatgtc - <u>negative</u>

OT Site 7 CCAGGATAATATCCAATGTGTAG chr16: 34645325-34645347 screened by SURVEYOR tgctcactgctgataggtctg ggatatcattggaccccaca - <u>negative</u>

OT Site 8 TTAGGGTAATATCCAATGTGAGG chr3: 89806272-89806294 screened by SURVEYOR tatctcggcccaagaagaag caaagaccaggatcccaatg - <u>negative</u>

OT Site 9 CAATAATCATCTCCAATGTGCAG chr4: 164282994-164283016 screened by SURVEYOR agcaggtcttcagcttggtt ccagaggccctcaaattaca - <u>negative</u>

OT Site10 CGAAGGTCCACTCCAATGTGCAG chr17: 19811676-19811698 screened by SURVEYOR tcgtgggaaggaaagacttg ggcagtccatgcctgtttat – <u>negative</u>

Methods: brain slice preparation, electrophysiology, protein quantification and computational modelling

Brain slice preparation

Rats were killed, the brains removed, and hippocampi dissected and sliced in ice-cold sucrose-based solution containing (in mM): 205 Sucrose, 10 Glucose, 26 $NaHCO_3$, 2.5 KCl, 1.25 NaH_2PO_4, 0.5 $CaCl_2$, 5 $MgSO_4$, saturated with 95% O_2 and 5% CO_2. Transverse 400 µm hippocampal slices were cut using the Leica LS1200 vibratome. Slices were incubated in artificial cerebrospinal fluid (aCSF) at 35 °C for 30 min and then at room temperature for 30 min. aCSF contained (in mM): mM: 124 NaCl, 3 KCl, 24 $NaHCO_3$, 1.25 NaH_2PO_4 10 Glucose, 2.5 $CaCl_2$, 1.3 $MgSO_4$, saturated with 95% O_2 and 5% CO_2.

Whole-cell patch-clamp recordings

The recording chamber was perfused with oxygenated aCSF at 32 °C. Slices were visualised using a differential interference contrast Scientifica SliceScope microscope. Borosilicate glass pipettes (pipette resistance of 4-7 MΩ) were pulled using a horizontal P-97 Sutter-instruments puller. 3 different internal solutions were used, potassium-based, potassium-based with Qx-314, and caesium-based. They contained (in mM):

Potassium-based: 120 KMeSO3, 8 NaCl, 10 HEPES, 4 Mg-ATP, 0.3 Na-GTP, 0.2 EGTA, 10 KCl, ~295 mOsm, 7.4 pH

Potassium-based with QX-314: 120 KMeSO3, 8 NaCl, 10 HEPES, 4 Mg-ATP, 0.3 Na-GTP, 0.2 EGTA, 10 KCl, 1 QX-314Cl, ~295 mOsm, 7.4 pH

Caesium-based: 130 CsMeSO3, 4 NaCl, 10 HEPES, 0.5 EGTA, 10 TEA, 2 Mg-ATP, 0.5 Na-GTP, 1 QX-314Cl, ~290 mOsm, 7.4 pH.

Neurobiotin (1 mg/mL) was added to the internal solution used in some intrinsic properties experiments for post hoc cell morphology investigation.

Recordings were obtained using a Multiclamp 700A Molecular Devices amplifier, filtered at 2.4 kHz, and sampled at 10, 20, or 25 kHz, depending on experiment, using a Cambridge Electronic Designs Micro 1401 data acquisition board. Cambridge Electronic Designs Signal 5.12 and Spike 2.7 software were used for data acquisition. All data are presented without adjustment for the junction potential (~−15 mV).

Synaptic stimulation and plasticity protocols

All recordings were made in the presence of picrotoxin 50 µM. Schaffer collateral (SC) and temporoammonic (TA) fibres were alternatively stimulated using a paired pulse protocol.

Experiment: AMPA:NMDA ratio

Using the caesium-based internal solution, cells were held at -70 mV for 10 min, +40 mV for 5 min, and -70 mV for another 10 min. This allowed for the acquisition of predominantly AMPA-mediated EPSCs at -70 mV and combined AMPA- and NMDA-mediated EPSCs at +40 mV. AMPA-mediated EPSCs were measured at the peak amplitude, whilst NMDA-mediated EPSCs were measured at 45 ms after the first stimulation artefact. If the AMPA-mediated EPSCs recorded before and after the voltage switch differed by more than 30%, data was excluded from the analysis.

Experiment: AMPA mEPSC

mEPSCs were recorded using the caesium-based internal solution. Recordings were made in the presence of TTX 500 nM and at a holding potential of -65 mV. 10 µM NBQX was applied in a subset of the initial experiments to confirm that the mEPSCs were AMPA-mediated (Fig S5). The following parameters were used for mEPSC detection post hoc: -3 pA amplitude, 0.1 ms tau (rise), 3 ms tau (decay), dead time 7 ms, rising edge window 2 ms.

Experiment: SK-mediated modulation of NMDAR

With a potassium-based QX-314 internal solution, cells were held in current-clamp with enough current injection to keep them at approximately -50 mV. The aCSF contained CGP55845 1µM throughout the experiment, with subsequent applications of apamin 100 nM alone and together with and D-APV 50 µM for 10 min each. Compound EPSPs were elicited using a burst of 5 stimulations at 100 Hz. EPSP decay was modelled using a single exponential function fit between the linear component of the decay of the final EPSP and baseline, from the averaged traces of the final 3 min of every condition.

Experiment: GluN2b-mediated EPSC decay

With a caesium-based internal solution, cells were held at +40 mV. aCSF contained NBQX 10 µM throughout the experiment, with subsequent additions of RO256981 1 µM alone and in combination with D-APV 50 µM for 10 min each. The decay of the second EPSC was modelled using a double exponential function fit between the amplitude peak and the baseline, from the averaged traces of the final 3 min of every condition.

Experiment: Paired theta burst LTP in SC synapses

Using a potassium-based internal solution, SC and TA fibres were alternatively stimulated in the voltage clamp configuration at -65 mV. With a 5 min baseline and within 10 min of breaking into the whole-cell configuration, LTP was induced in the SC synapses using a 2 second paired theta burst protocol (5 Hz bursts of 5 stimuli at 100 Hz) with simultaneous presynaptic stimulation and somatic depolarization (2 ms, 1 nA) steps. LTP induction was performed in the current-clamp configuration with enough current injection to maintain the cell at -65 mV. LTP was measured at 25-30 min post induction, using the TA pathway as a negative control.

Experiment: Associative LTP in SC *and TA synapses*

Using a potassium-based internal solution, the cell was held at -65 mV. SC (from either side of the recording cell) and TA fibres were alternatively stimulated. Prior to aLTP induction,

stimulation strength was adjusted to produce EPSCs with a mean amplitude of ~100 pA. With a 5 min baseline and within 10 min of breaking into the whole-cell configuration, LTP was induced in the one of the SC and the TA synapses using a 2 s theta burst (5 Hz) protocol with simultaneous presynaptic stimulation of the SC and the TA fibres, with no somatic depolarization steps. The SC pathway from the other side of the recording cell (the one that did not participate in LTP induction) was used as a control pathway. The cell was maintained at -55 mV in current clamp during induction and the theta burst train was repeated for a total of 3 times, at a 20 s interval. Initial baseline EPSC amplitudes were adjusted to be approximately 100 pA. LTP was measured at 25-30 min post induction. A pathway check was run at the end of the experiment to ensure the synapses stimulated belonged to distinct pathways (Fig S6). The experiment was repeated in the presence of 77-LH-28-1 7 µM, with baseline EPSC amplitudes adjusted to be approximately 100 pA. In the induction trace analysis, spikes were identified by finding local maxima over -20 mV. 2 ms before and 4 ms after the identified peaks, segments of the traces were removed. The missing values were interpolated to produce an approximation of the underlying EPSP.

Experiment: Supralinear integration of dendritic spiking into plateau potentials

Using a potassium-based internal solution containing QX-314, a single and a compound EPSP (burst of 5 stimulations at 100 Hz) in the SC pathway were recorded using increasing stimulus intensity in the presence of CGP55845 1µM. The subthreshold rising EPSP slope of the single EPSP was used to normalise area under the curve comparisons of the compound EPSP across conditions. The area under the curve was calculated from 50 ms to 450 ms after the compound EPSP. The slope-area relationship was smoothed using Savitzky–Golay filtering. The data was then passed through a change point analysis function in Matlab designed to find changes in signal to identify the threshold of nonlinearity [1]. The maximum recorded area under the curve (AUC) and its corresponding single EPSP slope was measured as a ratio to allow between-subject comparisons. D-APV 50 µM was applied at the end of the experiment

during ongoing maximal stimulation. The experiment was repeated in the presence of 4AP 0.3 mM, CP339818 5 µM, carbachol 1 µM, or 77-LH-28-1 7 µM.

Experiment: Intrinsic cell properties

Using a potassium-based internal solution, sometimes containing 1 mg/mL neurobiotin, intrinsic cell properties were measured in a specific order. Immediately after breaking in whole-cell, the clamping configuration was changed to I=0 to record the resting membrane potential (RMP). Subsequently, the current clamp configuration was adopted for rheobase experiments and enough current was injected to keep the cell at -65 mV. Depolarising 0.8 s current steps of variable magnitude were applied until a single action potential was fired consistently. A chirp current injection followed, 40 pA peak to peak and 20 s in duration, increasing in frequency from 0.2 to 20 Hz, to allow the measurement of impedance and resonance. Subsequently, hyperpolarising and depolarising steps (-150 to 250 pA and 0.8 s). 20 µM ZD-7288 was then washed on, followed by a repeat of the current step and chirp current injection protocols. Impedance was calculated by first applying the fast Fourier transform algorithm to the input current and output voltage sinusoid chirp data and then by taking their complex ratio. Savitzky–Golay filtering was used to smooth the data.

Immunohistochemistry and morphological analysis

Brain slices were placed in 4% PFA for 24-40 hours and then kept in phosphate buffered saline (PBS) (4 °C). Slices were washed in PBS, permeabilised in TritonX100 1:100 in PBS for 1 h, washed and incubated in PBS with 3% bovine serum albumin (BSA) 1 h. Alexafluor-594-streptavidin (1:1000) in PBS with 3% BSA was applied to bind to neurobiotin. Slices were washed in PBS with 3% BSA and incubated with DAPI (1:1000) in PBS with 3% BSA. Slices were then mounted onto glass slides. The slices were imaged using a widefield fluorescence microscope, using Leica LAS X software. The acquired images were processed using Fiji imageJ 1.8.0 software. The publicly available Simple Neurite Tracer plugin was used for semi-

automated tracing, visualisation, and analysis of the images [2]. The Sholl Analysis extension of the Simple Neurite Tracer plugin was also employed [3].

Protein Quantification

Western blot analysis was conducted on hippocampal tissue from wt (n=12) and het (n=12) animals. Each tissue sample was lysed in Syn-PER lysis and extraction buffer (Thermo Fisher, UK) with mini protease inhibitor cocktail (Roche Diagnostics) and phosphatase inhibitor (Cell signalling, UK) according to description from manufacturer. After using BCA Assay kit to measure the total amount of protein in each sample, electrophoresis and blotting were carried out. Gels (4–12% NuPAGE Bis-Tris Midi, 45 well) were loaded with 40ug of protein was loaded per well. Samples were added to Laemmli buffer at a 1:1 ratio and this mixture heated at 96 ^{o}C for 5 minutes to denature protein-protein interactions and facilitate antibody bindings. Samples were arranged so that genotypes were counterbalanced across gels with a WT standard used on each gel for comparison. Gels were run at room temperature in NuPAGE^{TM} Running Buffer (Invitrogen, UK) at 85V for 20 mins and then for a further hour at 115V. Protein was then transferred to 0.45 um pore size nitrocellulose membrane (Invitrogen, UK) at 85 V for 2 hours 15 minutes at an ambient temperature of 4 ^{o}C in NuPAGE^{TM} Transfer Buffer (Invitrogen, UK) containing 10% 2-propanol (ThermoFisher Scientific, UK). Membranes containing transferred protein were washed in Tris-Buffered Saline (20mM Tris, 150mM NaCl, pH 7.6) with 0.1% Tween 20 (TBST) before blocking in 5% milk for one hour at room temperature with gentle rocking. Primary antibodies (rabbit anti-PSD93, 1:1000, Cell Signalling Technology, USA; mouse anti-GluN1, 1:1000, Merck Millipore, UK; rabbit anti-PSD95, 1:2000, Abcam, UK; mouse anti-GAPDH, 1:5000, Abcam, UK) were diluted to appropriate concentrations in 5% milk and incubated with the membrane overnight at 4 ^{o}C. Membranes were then subject to 3 x 10 minute TBST washes before incubation with the appropriate fluorescent IRDye 680RD secondary antibodies at 1:15,000 dilution in 5% milk at room temperature. After another series of TBST washes membranes were imaged on Odyssey CLx Imaging System (Li-COR, Germany). Densiometric analysis of bands was

performed using ImageLab 6.0. The densities (with background subtracted) of the protein of interest were divided by the loading control densities for each sample to provide normalised values. Densities were then averaged by group.

Computational modelling

All modelling work was done using the NEURON simulation environment [4]. In-house morphological reconstructions of wt and het neurons were used. A total of 6 representative reconstructions (3 wt, 3 het) were populated with mechanisms corresponding to Ka [5-6], Kir [7-9], Km [10], Kdr [5], and NaV [5,10] channels with parameter values tuned to replicate experimental data to assess input resistance in relation to morphology (Table S2). The channel mechanisms were taken from the Neuron Model database [11]. To compare the contributions of the different candidate channels to input resistance, a wt reconstruction was populated with leak current and Ka or Kir channel mechanisms, whose conductance was scaled by 0.5-5. To assess the contribution of input resistance and potassium channels on dendritic integration, the following series of steps were performed. Dendrites were assigned a number which was then shuffled (the same seed was used across conditions). Dendrites were cumulatively recruited using a glutamate mechanism (1 synapse per dendrite), with the ratio of apical proximal and apical tuft dendrites systematically iterated upon. This process was then repeated following changes to conditions, such as the inclusion of a potassium channel mechanism. All dendritic integration simulations were done on the representative wt reconstruction. Model parameters are summarised in Table S3 [12-14].

Table S2 peak channel conductances used in computational modelling simulations

**Peak conductance (S/cm^2)**		
**Channel**	Soma	Dendrites
**Km**	0.017	0.017
**NaV**	0.1	0.03
**Kdr**	0.04	0.04
**Ka**	0.02	0.02
**Kir**	0.0000144	0.0000144
**Leak (gpas)**	0.000025	0.000025

Km M-type potassium channel

NaV Voltage-gated sodium channel

Kdr Delayed-rectifier potassium channel

Ka A-type potassium channel

Kir Inwardly-rectifying potassium channel

Gpas passive conductance

Table S3 model parameters used in computational modelling simulations

**Model parameters**	
**Axial resistivity (Ω cm)**	150
**Membrane capacitance (μf/cm2)**	1.5
**Membrane resistivity (kΩ cm2)**	40
**Resting membrane potential (mV)**	-65
**Temperature (°C)**	35
**Equilibrium potential (K) (mV)**	-90
**Equilibrium potential (Na) (mV)**	55
**NMDA rise time constant (ms)**	4
**NMDA decay time constant (ms)**	42
**Equilibrium potential (NMDA) (mV)**	0
**AMPA rise time constant (ms)**	0.5
**AMPA decay time constant (ms)**	1.5

Figures

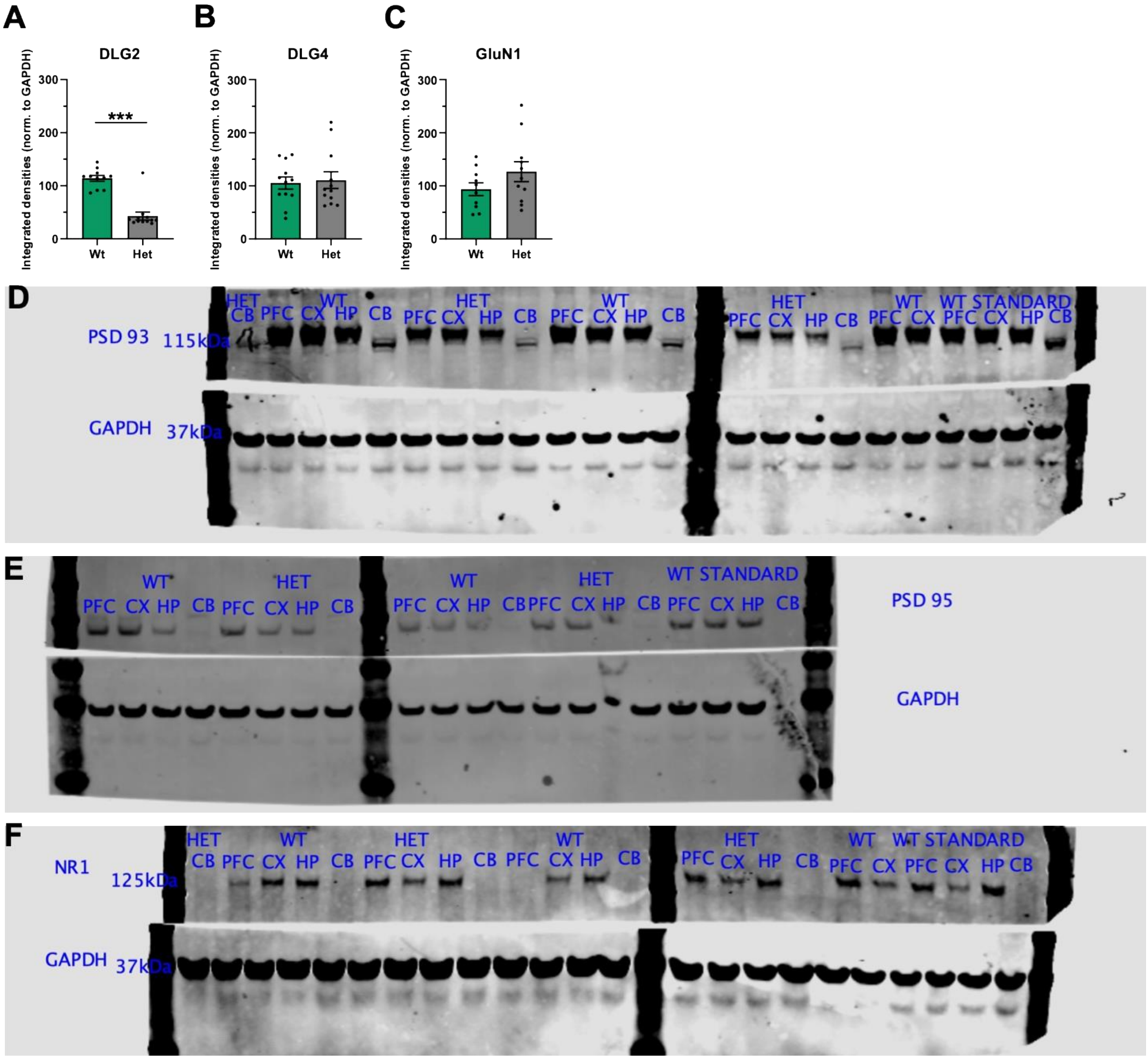

Figure S3 DLG2 expression is reduced in *Dlg2+/-* hets, with no change in DLG4 or GluN1 expression. Western blot analysis for expression of DLG2 (unpaired t-test: $P < 0.001$) **(A)**, DLG4 (unpaired t-test: $P = 0.784$) **(B)**, and GluN1 (unpaired t-test: $P = 0.163$) **(C)** in the hippocampus of *Dlg2+/-* het and wt rats. Data from 23 (DLG2), 24 (DLG4), and 21 (GluN1) rats. **D-F)** Example Western blots in Dlg2+/- het rat hippocampus, cerebellum, cortex, and prefrontal cortex. Expression of DLG2 (PSD93) **(D)**, DLG4 (PSD95) **(E)**, and GluN1 (NR1) **(F)** in the hippocampus (HP), prefrontal cortex (PFC), cortex (CX), and cerebellum (CB) of Dlg2+/- het and wt rats**.** Summary values depicted as mean ± SEM. * $P < 0.05$, ** $P < 0.01$, *** $P < 0.001$ (unpaired t-test)

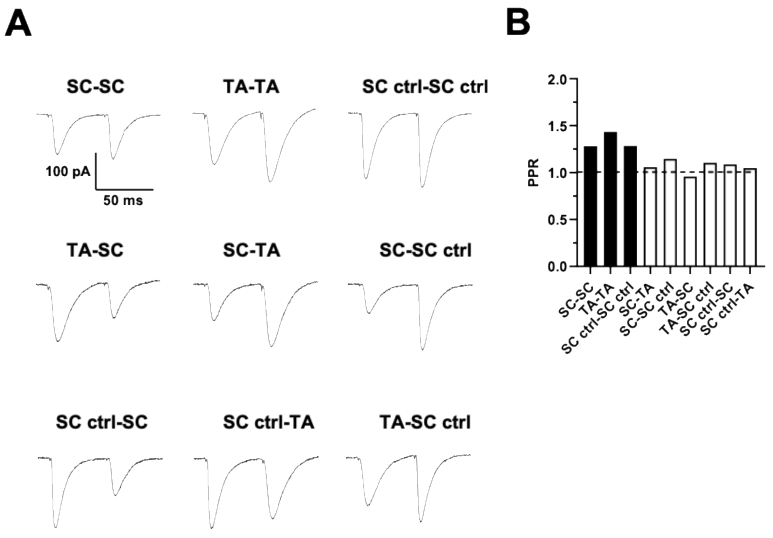

Figure S4 Example pathway independence check from the aLTP experiments. **A)** EPSC traces of 3 pathway responses recorded in different combinations, used to calculate paired-pulse ratio. **B)** Paired-pulse ratios corresponding to the traces in panel A. When the same pathway is stimulated in sequence, there is facilitation. When different pathways are stimulated in sequence, there is little to no facilitation

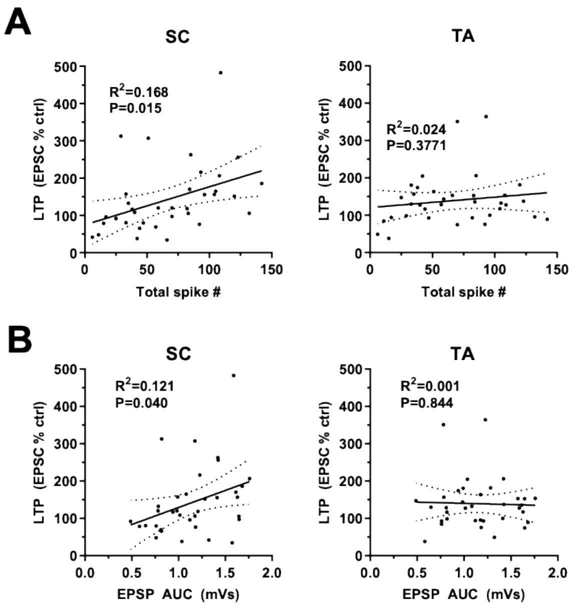

Figure S5 Spikes and AUC are positively correlated with aLTP in the SC but not the TA pathway. Data from the 3-pathway aLTP experiment. **A)** Correlation between total spike number and LTP in the SC (Pearson correlation: R^2 = 0.168, P = 0.015) and TA (Pearson correlation: R^2 = 0.024, P = 0.377) pathways. **B)** Correlation between EPSP AUC and LTP in the SC (Pearson correlation: R^2 = 0.121, P = 0.040) and TA (Pearson correlation: R^2 = 0.001, P = 0.844) pathways.

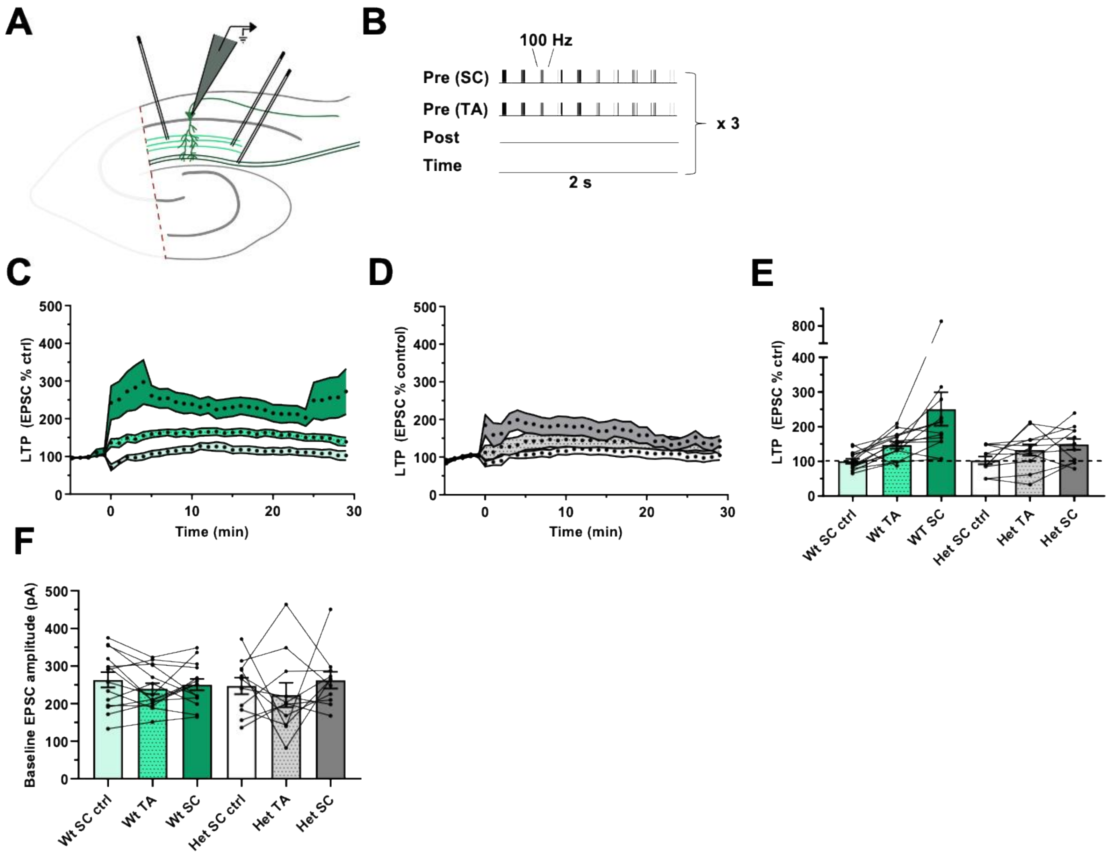

Figure S6 Increased baseline EPSC amplitude produces aLTP in both genotypes. **A)** Schematic representation of the hippocampal slice recording setup, with the CA3 removed and stimulating electrodes in two separate areas of the stratum radiatum and in the stratum lacunosum moleculare. **B)** aLTP induction protocol, where one SC pathway and one TA pathway were tested and where the second SC pathway acted as a negative control. There was no induced somatic depolarisation. This induction protocol was repeated thrice at an interval of 10 seconds. aLTP over time in wts **(C)** and *Dlg2+/-* hets **(D)**. **E)** aLTP at the 25-30 minute mark post induction across genotype and pathway (3-way repeated-measures ANOVA: pathway effect: $F_{2, 36} = 6.602$, $P = 0.004$. Genotype main effect: $F_{1, 28} = 0.064$, $P = 0.803$. Genotype x pathway interaction: $F_{2, 36} = 0.543$, $P = 0.586$). **F)** Baseline EPSC amplitude across genotype (3-way repeated-measures ANOVA: genotype x aspect interaction: $F_{1, 18} = 0.144$, $P = 0.709$. Genotype x aspect x pathway interaction: $F_{2, 36} = 0.193$, $P = 0.825$). Hets: 11 cells, 9 animals and wts: 14 cells, 8 animals. Summary values depicted as mean ± SEM. * $P < 0.05$, ** $P < 0.01$, *** $P < 0.001$ (3-way ANOVA between subject effect)

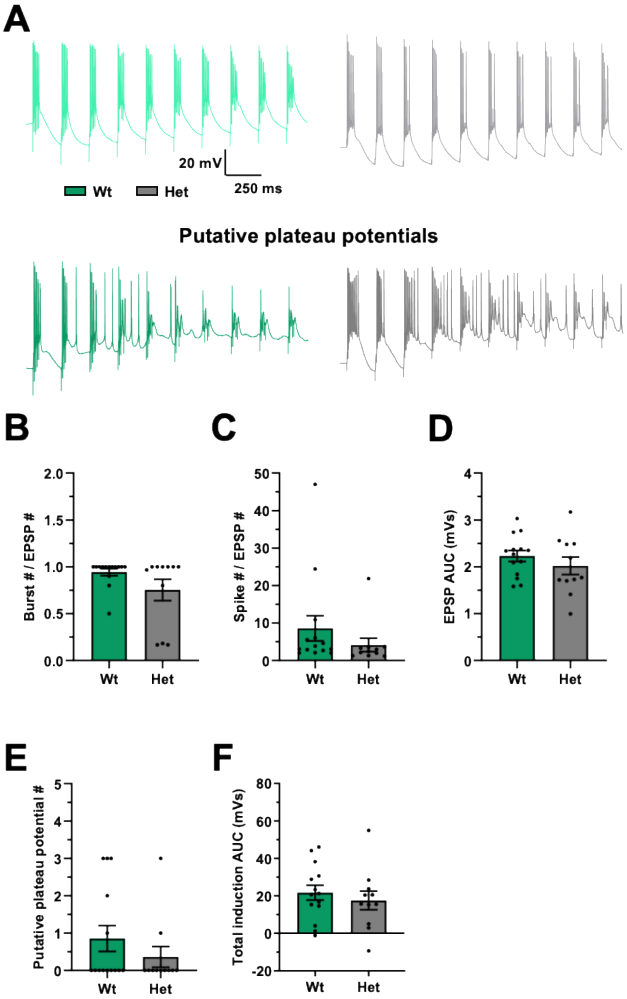

Figure S7 Increased baseline EPSC amplitude facilitates putative plateau potential generation and aLTP. **A)** example aLTP induction traces showing putative plateau potentials. Burst number (3-way ANOVA: genotype main effect: $F_{1, 25} = 1.781$, $P = 0.199$) **(B)**, spike number (3-way ANOVA: genotype main effect: $F_{1, 25} = 0.000$, $P = 0.985$) **(C)**, mean EPSP area under the curve (AUC) (3-way ANOVA: genotype main effect: $F_{1, 25} = 0.478$, $P = 0.498$) **(D)**, putative plateau potential number (3-way ANOVA: genotype main effect: $F_{1, 25} = 0.007$, $P = 0.936$) **(E)**, total induction AUC (3-way ANOVA: genotype main effect: $F_{1, 25} = 0.167$, $P = 0.688$) **(F)** across genotype. Hets: 11 cells, 9 animals and wts: 14 cells, 8 animals. Summary values depicted as mean ± SEM. * $P < 0.05$, ** $P < 0.01$, *** $P < 0.001$ (3-way ANOVA between subject effect)

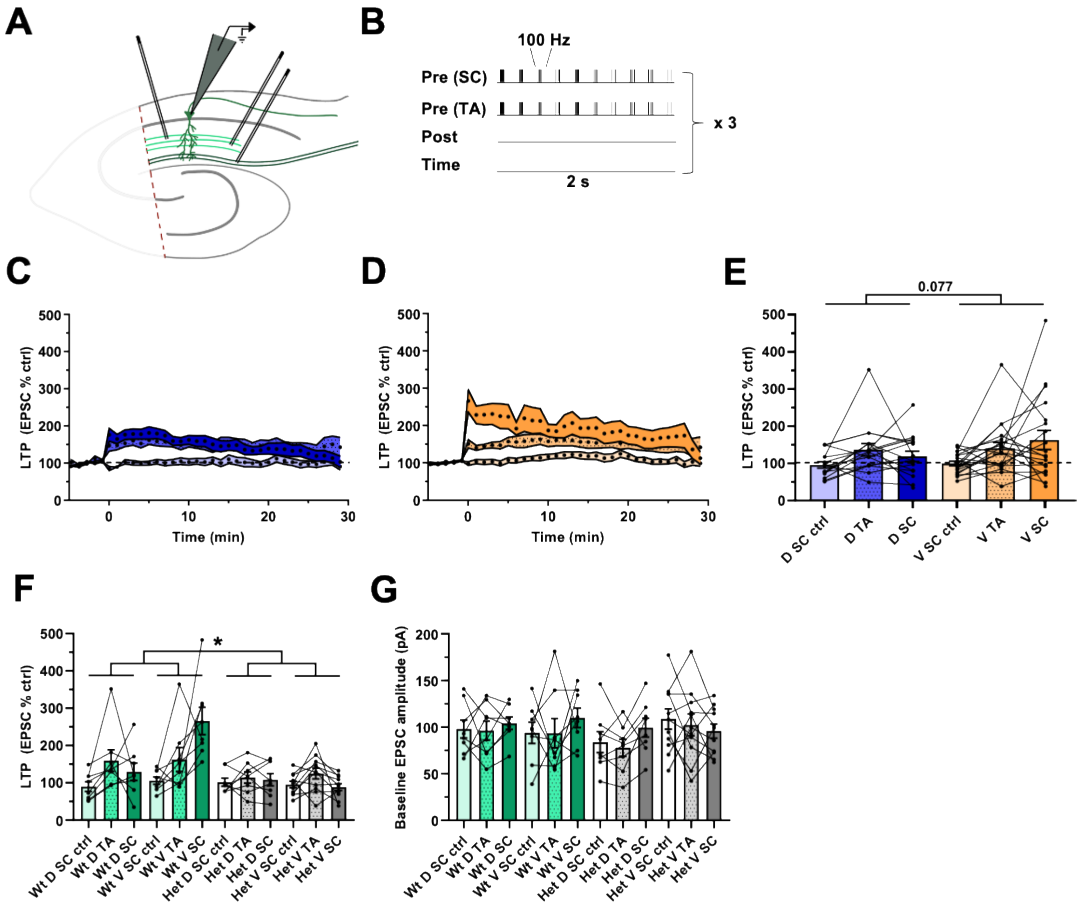

Figure S8 Attenuated aLTP in the dorsal hippocampus. **A)** Schematic representation of the hippocampal slice recording setup, with the CA3 removed and stimulating electrodes in two separate areas of the stratum radiatum and in the stratum lacunosum moleculare. **B)** aLTP induction protocol, where one SC pathway and one TA pathway were tested and where the second SC pathway acted as a negative control. There was no induced somatic depolarisation. This induction protocol was repeated thrice at an interval of 10 seconds. aLTP over time in dorsal **(C)** and ventral **(D)** aspects of the hippocampus. **E)** aLTP at the 25-30 minute mark post induction across dorsal-ventral aspects of the hippocampus and pathways (3-way repeated-measures ANOVA: pathway effect: $F_{2, 54} = 7.300$, $P = 0.002$. Aspect main effect: $F_{1, 27} = 3.374$, $P = 0.077$. Aspect x pathway interaction: $F_{2, 54} = 2.598$, $P = 0.084$). **F)** aLTP at the 25-30 minute mark post induction across genotype, aspects of the hippocampus, and pathway (3-way repeated-measures ANOVA: genotype x aspect interaction: $F_{1, 27} = 4.510$, $P = 0.043$. Genotype x aspect x pathway interaction: $F_{2, 54} = 6.195$, $P = 0.004$). **G)** Baseline EPSC amplitude across genotype (3-way repeated-measures ANOVA: genotype x

aspect interaction: $F_{1, 27} = 1.577$, $P = 0.220$. Genotype x aspect x pathway interaction: $F_{2, 54} = 1.744$, $P = 0.185$). Hets: 19 cells, 9 animals and wts: 16 cells, 8 animals. Summary values depicted as mean ± SEM. * $P < 0.05$, ** $P < 0.01$, *** $P < 0.001$ (3-way ANOVA between subject effect)

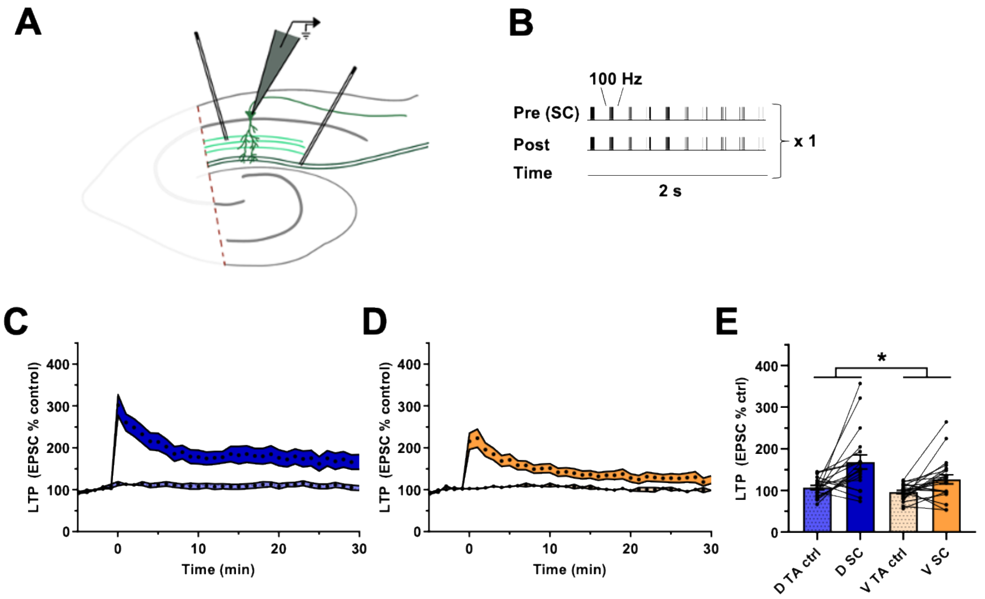

Figure S9 Attenuated theta burst LTP in the ventral aspect of the hippocampus. **A)** Schematic representation of the hippocampal slice recording setup, with the CA3 removed and stimulating electrodes in two separate areas of the stratum radiatum and in the stratum lacunosum moleculare. **B)** Theta burst LTP induction protocol, where the SC pathway was paired with somatic depolarisation and where the TA pathway acted as a negative control. Theta burst LTP over time in doral **(C)** and ventral **(D)** aspects of the hippocampus. **E)** Theta burst LTP at the 25-30 minute mark post induction across genotype (3-way repeated-measures ANOVA: pathway effect: $F_{1, 33} = 18.979$, $P < 0.001$. Aspect main effect: $F_{1, 33} = 5.259$, $P = 0.028$. Aspect x pathway interaction: $F_{1, 33} = 2.085$, $P = 0.158$). Hets: 21 cells, 9 animals and wts: 20 cells, 10 animals. Summary values depicted as mean ± SEM. * $P < 0.05$, ** $P < 0.01$, *** $P < 0.001$ (3-way ANOVA between subject effect)

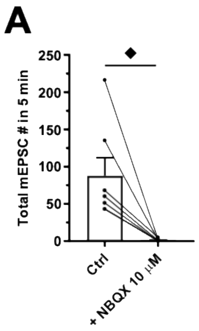

Figure S10 mEPSC are blocked by NBQX. **A)** Total mEPSCs before and after the application of NBQX 10 µM. Summary values depicted as mean ± SEM. ♦ $P < 0.05$, ♦♦ $P < 0.01$, ♦♦♦ $P < 0.001$ (paired t-test)

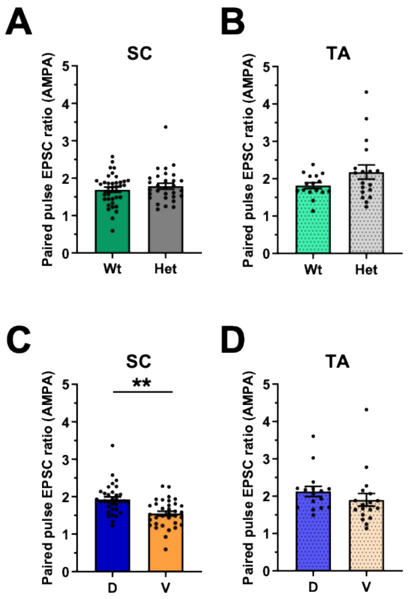

Figure S11 Paired pulse facilitation (AMPA/NMDA ratio experiment). Paired pulse EPSC ratio, taken from the AMPA component at -70 mV, across genotype in the SC (3-way ANOVA: genotype main effect: $F_{1, 67} = 0.010$, $P = 0.921$) **(A)** and TA (3-way ANOVA: genotype main effect: $F_{1, 35} = 1.739$, $P = 0.198$) **(B)** pathways. The same measurement but across dorsal/ventral aspects of the hippocampus in the SC (3-way ANOVA: aspect main effect: $F_{1, 67} = 11.367$, $P = 0.001$) **(C)** and TA (3-way ANOVA: aspect main effect: $F_{1, 35} = 0.087$, $P = 0.770$) **(D)** pathways. Hets: 30 cells, 18 animals and wts: 38 cells, 19 animals for the SC data set and hets: 18 cells, 9 animals and wts: 16 cells, 8 animals for the TA data set. Summary values depicted as mean ± SEM. * $P < 0.05$, ** $P < 0.01$, *** $P < 0.001$ (3-way ANOVA between subject effect)

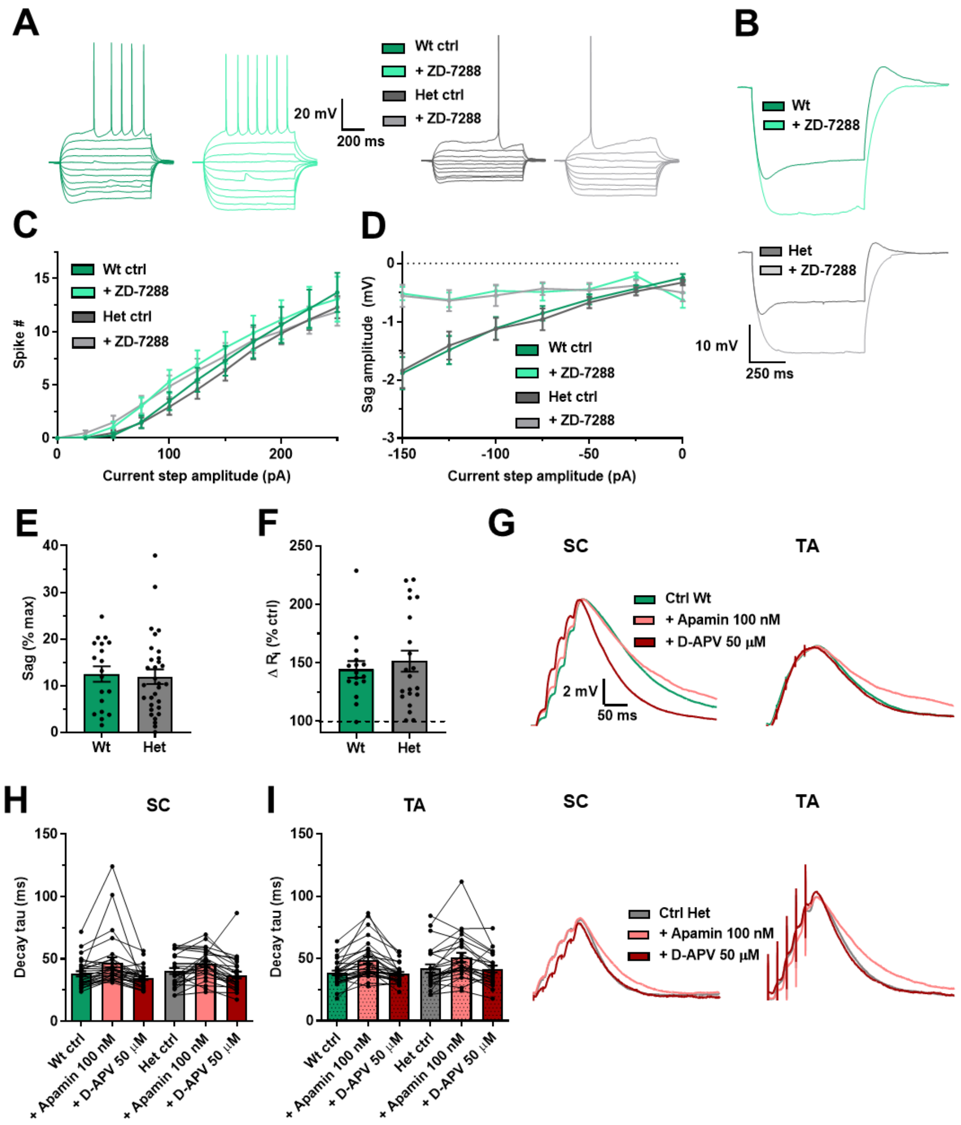

Figure S12 Unaltered HCN and SK channel function in the *Dlg2+/-* hets. **A)** Example voltage traces in response to current steps (-150 to 250 pA) before and after application of ZD-7288 20 µM. **B)** Voltage traces in response to a hyperpolarising current step of -150 pA before and after the application of ZD-7288 20 µM. Spike number (3-way repeated-measures ANOVA: drug effect: $F_{1, 28} = 0.321$, $P = 0.576$. Current step effect: $F_{10, 280} = 99.423$, $P < 0.001$. Genotype main effect: $F_{1, 28} = 0.272$, $P = 0.606$. Drug x genotype interaction: $F_{1, 28} = 0.304$, $P = 0.586$. Drug x step interaction: $F_{10, 280} = 4.126$, $P < 0.001$. Drug x step x genotype interaction: $F_{10, 280} = 0.134$, $P = 0.999$) **(C)** and sag amplitude (3-way repeated-measures ANOVA: drug effect: $F_{1, 28} = 14.797$, $P = 0.001$. Current step effect: $F_{6, 168} = 14.072$, $P < 0.001$. Genotype main effect: $F_{1, 28} = 0.03$, $P = 0.864$. Drug x genotype interaction: $F_{1, 28} = 0.206$, $P = 0.654$. Drug x step

interaction: $F_{10, 280} = 4.126$, $P < 0.001$. Drug x step x genotype interaction: $F_{10, 280} = 0.564$, $P = 0.758$) **(D)** across genotype and before and after the application of ZD-7288 20 µM. Sag as a percentage of max voltage deflection (3-way repeated-measures ANOVA: genotype main effect: $F_{1, 50} = 0.182$, $P = 0.672$) **(E)** and change in input resistance (3-way repeated-measures ANOVA: genotype main effect: $F_{1, 37} = 0.351$, $P = 0.558$) **(F)** following the application of ZD-7288 20 µM. Hets: 21 cells, 11 animals and wts: 16 cells, 7 animals. **G)** EPSPs before and after the application of apamin 100 nM. EPSP decay tau across genotype in SC (3-way repeated-measures ANOVA: drug effect: $F_{2, 92} = 18.327$, $P < 0.001$. Genotype main effect: $F_{1, 46} = 0.057$, $P = 0.812$. Drug x genotype interaction: $F_{2, 92} = 0.76$, $P = 0.471$.) **(H)**, and TA (3-way repeated-measures ANOVA: drug effect: $F_{2, 86} = 29.346$, $P < 0.001$. Genotype main effect: $F_{1, 43} = 0.391$, $P = 0.535$. Genotype x drug interaction: $F_{2, 86} = 0.166$, $P = 0.847$). **(I)** pathways. Hets: 26 cells, 8 animals and wts: 28 cells, 9 animals for the SC data set and hets: 23 cells, 8 animals and wts: 29 cells, 9 animals for the TA data set. Summary values depicted as mean ± SEM. * $P < 0.05$, ** $P < 0.01$, *** $P < 0.001$ (3-way ANOVA between subject effect)

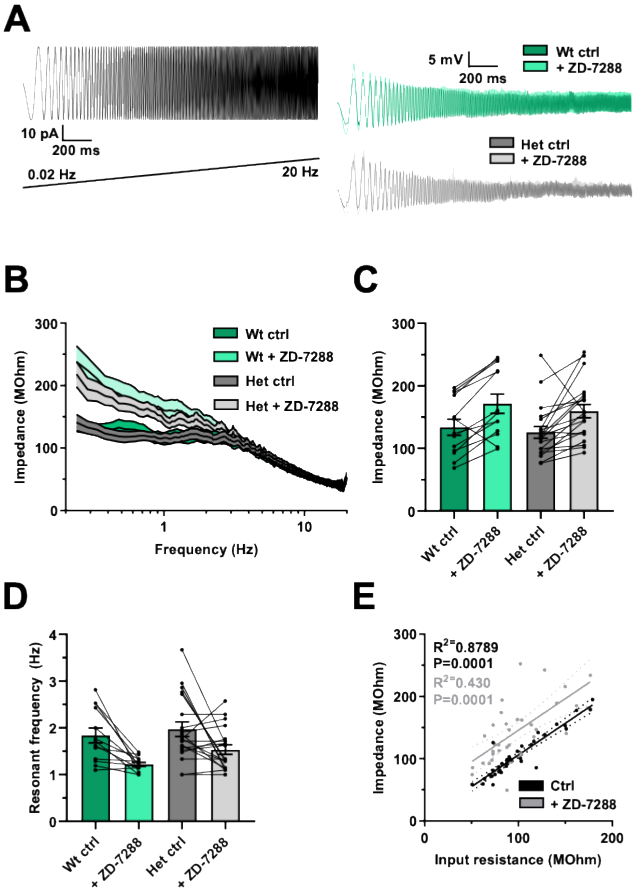

Figure S13 No effect of genotype on impedance and resonant frequency. **A)** input current wave (left) and output voltage waves (right) across genotype and before and after the application of ZD-7288 20 µM. **B)** impedance over 0.2-20 Hz input frequencies before and after the application of ZD-7288 20 µM across genotype. Maximum impedance in the 1-20 Hz range (3-way repeated-measures ANOVA: drug effect: $F_{1, 25} = 26.64$, $P < 0.001$. Genotype main effect: $F_{1, 25} = 0.044$, $P = 0.835$. Genotype x drug interaction: $F_{1, 25} = 0.033$, $P = 0.858$) **(C)** and the corresponding resonant frequency (3-way repeated-measures ANOVA: drug effect: $F_{1, 25} = 22.402$, $P < 0.001$. Genotype main effect: $F_{1, 25} = 1.639$, $P = 0.212$. Genotype x drug interaction: $F_{1, 25} = 1.538$, $P = 0.226$) **(D)**. **E)** Correlation between input resistance and impedance before (Pearson correlation: $R^2 = 0.879$, $P < 0.001$) and after (Pearson correlation: $R^2 = 0.430$, $P < 0.001$) application of ZD-7288 20 µM. Hets: 20 cells, 11 animals and wts: 13 cells, 6 animals. Summary values depicted as mean ± SEM. * P < 0.05, ** P < 0.01, *** P < 0.001 (3-way ANOVA between subject effect)

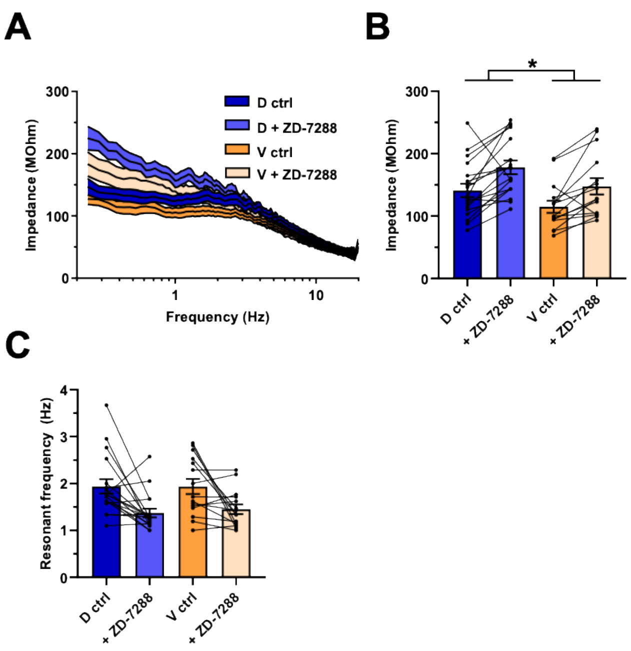

Figure S14 Reduced impedance in the ventral aspect of the hippocampus. **A)** impedance over 0.2-20 Hz input frequencies before and after the application of ZD-7288 20 µM across genotype. Maximum impedance in the 1-20 Hz range (3-way repeated-measures ANOVA: drug effect: $F_{1, 25} = 26.640$, $P < 0.001$. Aspect main effect: $F_{1, 25} = 6.300$, $P = 0.019$. Aspect x drug interaction: $F_{1, 25} = 0.427$, $P = 0.519$) **(B)** and the corresponding resonant frequency (3-way repeated-measures ANOVA: drug effect: $F_{1, 25} = 22.402$, $P < 0.001$. Aspect main effect: $F_{1, 25} = 0.584$, $P = 0.452$. Aspect x drug interaction: $F_{1, 25} = 0.055$, $P = 0.817$) **(C)**. Hets: 20 cells, 11 animals and wts: 13 cells, 6 animals. Summary values depicted as mean ± SEM. * $P < 0.05$, ** $P < 0.01$, *** $P < 0.001$ (3-way ANOVA between subject effect)

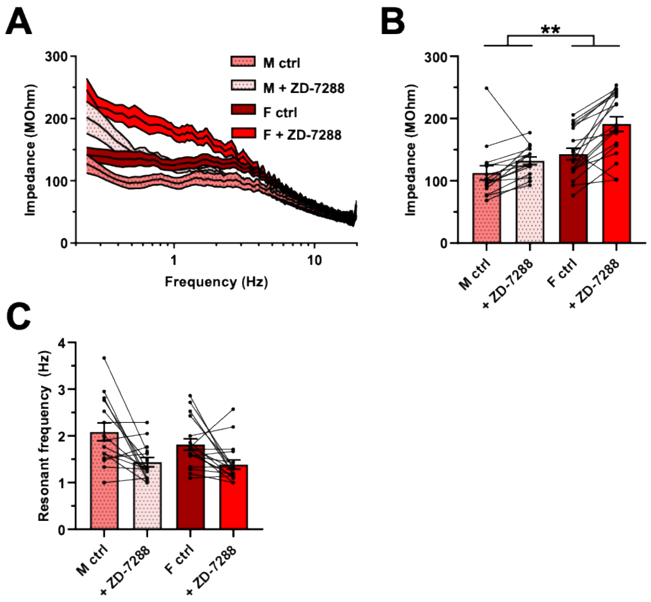

Figure S15 Increased impedance in neurons from female rats. **A)** impedance over 0.2-20 Hz input frequencies before and after the application of ZD-7288 20 µM across genotype. Maximum impedance in the 1-20 Hz range (3-way repeated-measures ANOVA: drug effect: $F_{1, 25} = 26.640$, $P < 0.001$. Sex main effect: $F_{1, 25} = 15.070$, $P = 0.001$. Sex x drug interaction: $F_{1, 25} = 2.943$, $P = 0.099$) **(B)** and the corresponding resonant frequency (3-way repeated-measures ANOVA: drug effect: $F_{1, 25} = 22.402$, $P < 0.001$. Sex main effect: $F_{1, 25} = 1.542$, $P = 0.226$. Sex x drug interaction: $F_{1, 25} = 1.078$, $P < 0.309$) **(C)**. Hets: 20 cells, 11 animals and wts: 13 cells, 6 animals. Summary values depicted as mean ± SEM. * $P < 0.05$, ** $P < 0.01$, *** $P < 0.001$ (3-way ANOVA between subject effect)

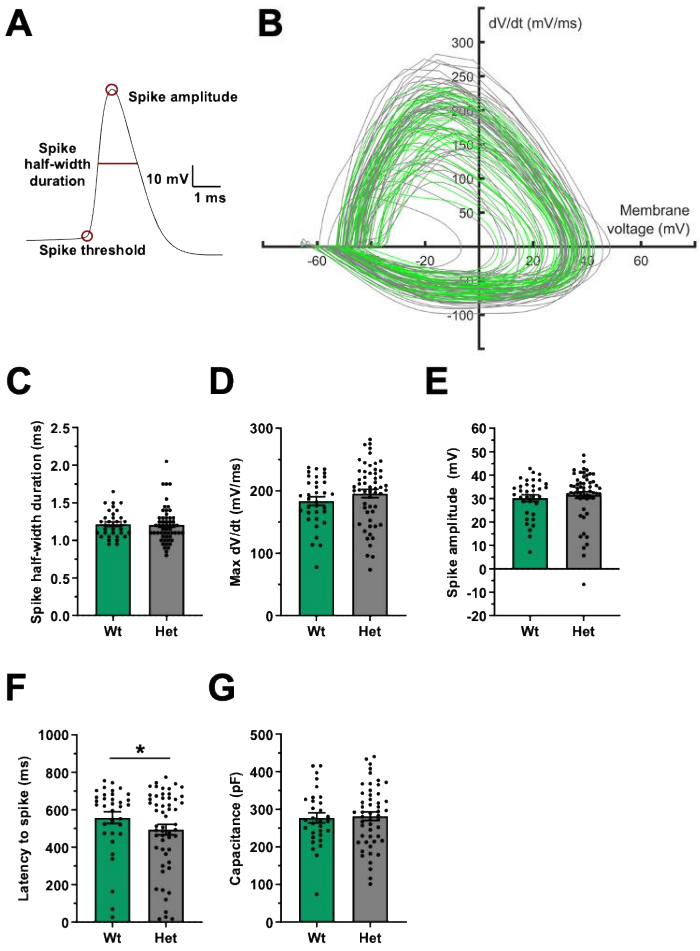

Figure S16 The effect of genotype on additional intrinsic properties. Data from the rheobase set of experiments. **A)** example spike from. **B)** Membrane voltage-spike dV/dT relationship across genotype (wt: green, *Dlg2+/-* het: gray). Spike half-width duration (3-way ANOVA: genotype main effect: $F_{1, 89} = 0.775$, $P = 0.381$) **(C)**, max dV/dt (3-way ANOVA: genotype main effect: $F_{1, 89} = 2.300$, $P = 0.133$) **(D)**, spike amplitude (3-way ANOVA: genotype main effect: $F_{1, 89} = 0.545$, $P = 0.462$) **(E)**, latency to spike (3-way ANOVA: genotype main effect: $F_{1, 89} = 4.122$, $P = 0.046$) **(F)**, and membrane capacitance (3-way ANOVA: genotype main effect: $F_{1, 82} = 0.053$, $P = 0.818$) **(G)** across genotype. Hets: 83 cells, 21 animals and wts: 53 cells, 17 animals. Summary values depicted as mean ± SEM. * $P < 0.05$, ** $P < 0.01$, *** $P < 0.001$ (3-way ANOVA between subject effect)

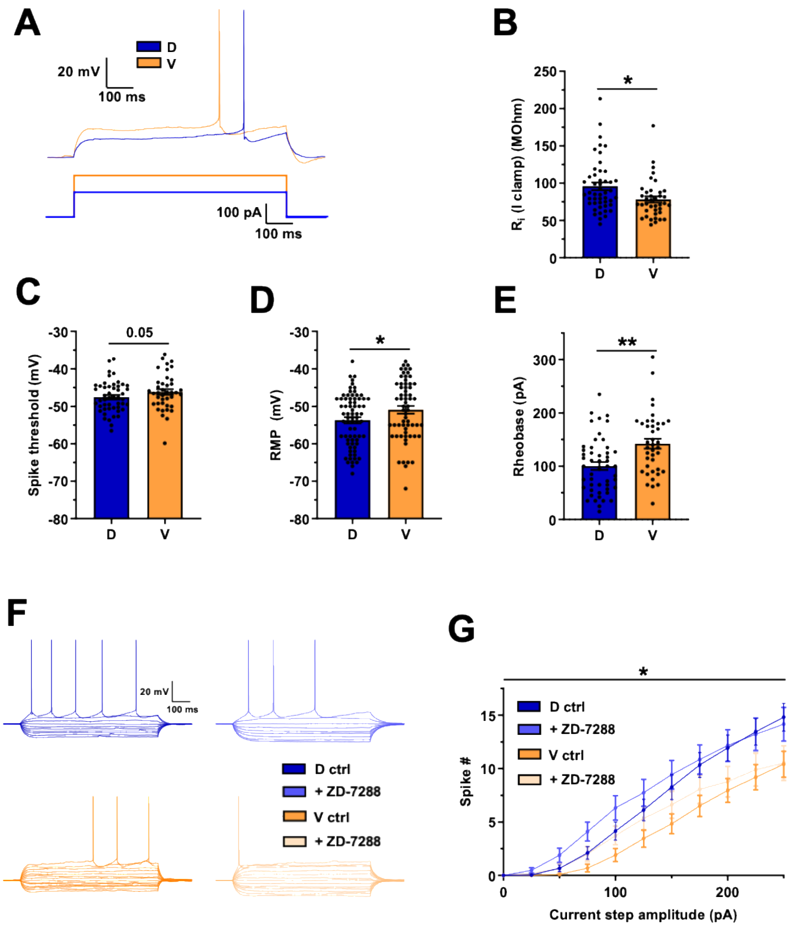

Figure S17 The effect of dorsal-ventral aspect of the hippocampus on intrinsic properties. Data from the rheobase set of experiments. **A)** Voltage deflections in response to a current step of equal size across genotype. Input resistance (3-way ANOVA: aspect main effect: $F_{1, 87}$ = 4.565, P = 0.036) **(B)**, spike threshold (3-way ANOVA: aspect main effect: $F_{1, 89}$ = 3.962, P = 0.05) **(C)**, resting membrane potential (RMP) (3-way ANOVA: aspect main effect: $F_{1, 136}$ = 4.722, P = 0.032) **(D)**, and rheobase (3-way ANOVA: aspect main effect: $F_{1, 90}$= 12.687, P = 0.001) **(E)**, across dorsal-ventral aspects of the hippocampus. Hets: 83 cells, 21 animals and wts: 53 cells, 17 animals. **F)** Example dorsal and ventral voltage traces in response to current steps (-150 to 250 pA) before and after application of ZD-7288 20 µM. Spike number across dorsal-ventral aspects of the hippocampus, before and after the application of ZD-7288 20 µM (3-way repeated-measures ANOVA: drug effect: $F_{1, 28}$ = 0.321, P = 0.576. Current step effect: $F_{10, 280}$ = 99.423, P < 0.001. Aspect main effect: $F_{1, 28}$ = 4.890, P = 0.035. Drug x aspect

interaction: $F_{1, 28} = 0.085$, $P = 0.773$. Drug x step interaction: $F_{10, 280} = 4.126$, $P < 0.001$. Drug x step x aspect interaction: $F_{10, 280} = 1.307$, $P = 0.226$) **(C)**. Hets: 21 cells, 11 animals and wts: 16 cells, 7 animals. Summary values depicted as mean ± SEM. * $P < 0.05$, ** $P < 0.01$, *** $P < 0.001$ (3-way ANOVA between subject effect)

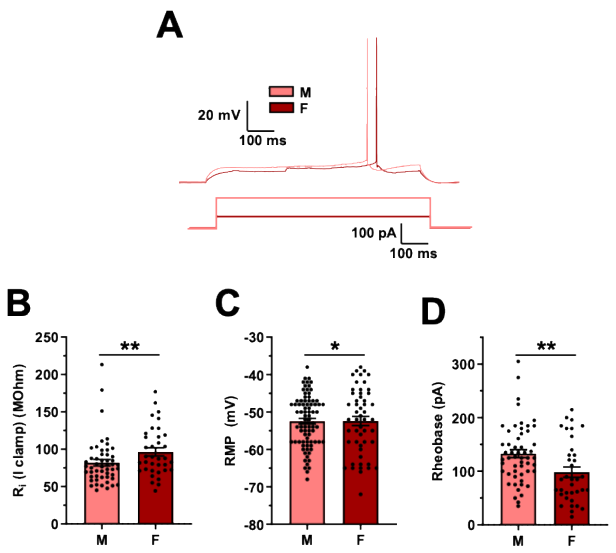

Figure S18 The effect of sex on intrinsic properties. Data from the rheobase set of experiments. **A)** Voltage deflections in response to a current step of equal size across genotype. Input resistance (3-way ANOVA: sex main effect: $F_{1, 87} = 11.739$, $P = 0.001$) **(B)**, resting membrane potential (RMP) (3-way ANOVA: sex main effect: $F_{1, 136} = 5.176$, $P = 0.025$) **(C)**, and rheobase (3-way ANOVA: sex main effect: $F_{1, 90} = 10.581$, $P = 0.002$) **(D)** across dorsal-ventral aspects of the hippocampus. Hets: 83 cells, 21 animals and wts: 53 cells, 17 animals. Summary values depicted as mean ± SEM. * $P < 0.05$, ** $P < 0.01$, *** $P < 0.001$ (3-way ANOVA between subject effect)

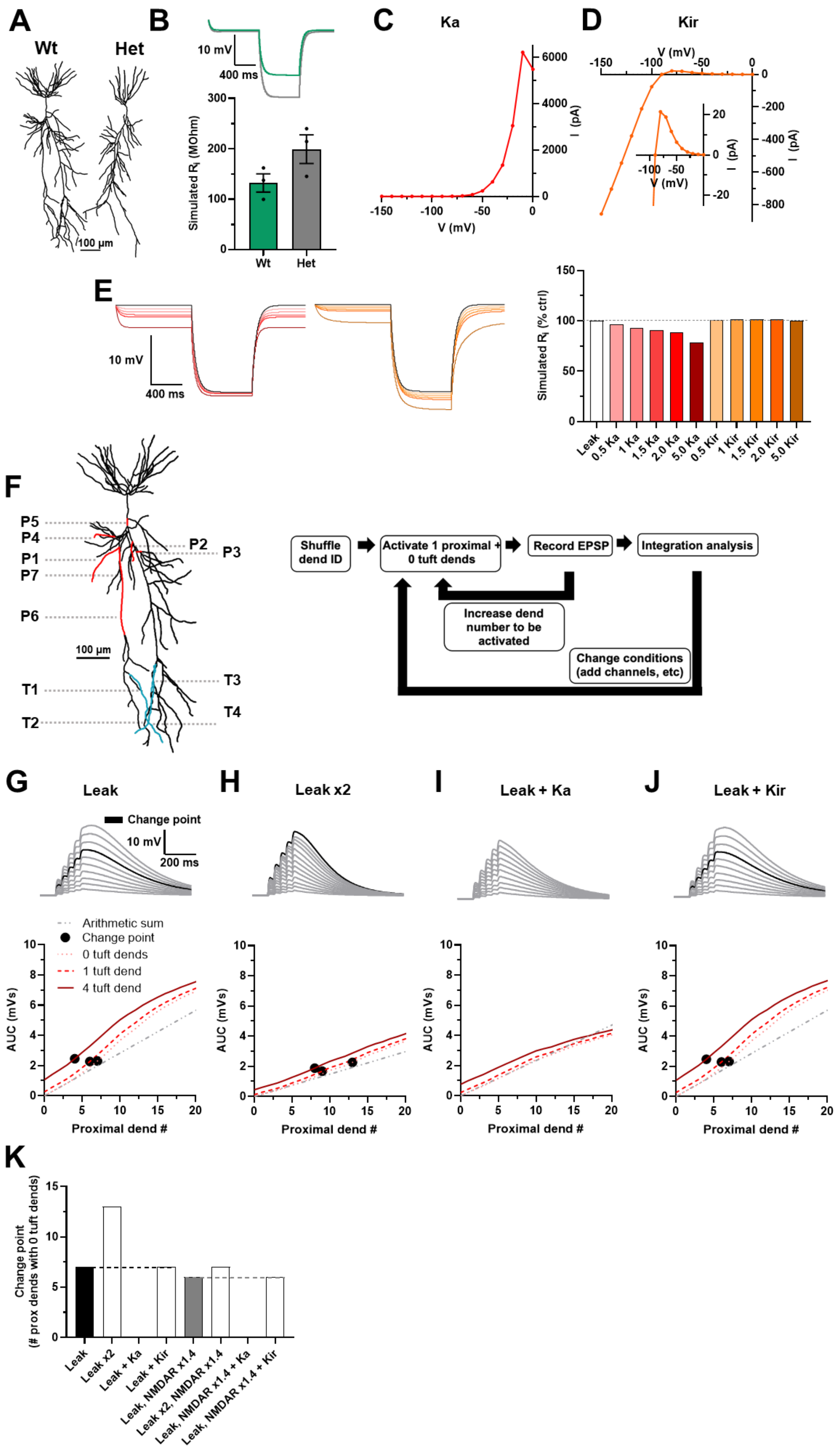
A
Wt
Het
100 µm
B
10 mV
400 ms
Simulated R_i (MOhm)
Wt
Het
C
Ka
V (mV)
I (pA)
D
Kir
V (mV)
I (pA)
E
10 mV
400 ms
Simulated R_i (% ctrl)
Leak
0.5 Ka
1 Ka
1.5 Ka
2.0 Ka
5.0 Ka
0.5 Kir
1 Kir
1.5 Kir
2.0 Kir
5.0 Kir
F
P5
P4
P1
P7
P6
P2
P3
100 µm
T1
T2
T3
T4
Shuffle dend ID
Activate 1 proximal + 0 tuft dends
Record EPSP
Integration analysis
Increase dend number to be activated
Change conditions (add channels, etc)
G
Leak
Change point
10 mV
200 ms
H
Leak x2
I
Leak + Ka
J
Leak + Kir
Arithmetic sum
Change point
0 tuft dends
1 tuft dend
4 tuft dend
AUC (mVs)
Proximal dend #
K
Change point (# prox dends with 0 tuft dends)
Leak
Leak x2
Leak + Ka
Leak + Kir
Leak, NMDAR x1.4
Leak x2, NMDAR x1.4
Leak, NMDAR x1.4 + Ka
Leak, NMDAR x1.4 + Kir

Figure S19 With equal ionic conductance, simulated het neurons have higher input resistance. Ka channels reduce input resistance and inhibit dendritic integration. **A)** Example reconstructions used in simulations. **B)** Simulated voltage traces in response to a -150 pA current step across genotype (top) and the corresponding input resistance (bottom). 6 neuronal reconstructions (3 wt, 3 het) were populated with Ka, Kir, Km, Kdr, NaV (voltage dependent potassium A-type, inward rectifier, M-type, delayed rectifier and sodium channels respectively) as well as a voltage independent leak conductance. Het reconstructed neurons showed consistently increased input resistance relative to wt supporting the conclusion that the *Dlg2+/-* hets have increased ionic conductances. Voltage-current relationships for Ka **(C)** and Kir **(D)** channels, illustrating outward- and inward-rectification, respectively. Inset in D shows the voltage-current relationship for Kir channels at a zoomed in scale for clarity. **E)** Voltage traces in response to a -150 pA current step in a wt reconstruction populated either with the Ka (pink) or the Kir (orange) channels (left). Channel conductance was scaled by a factor of 0.5-5 for each condition. Corresponding input resistance (right). These potassium channel selective effects on input resistance map onto the voltage-dependent activation curves of these channels and suggest an increase in Ka channel expression or function is a potential candidate to underly the decrease in input resistance in the *Dlg2+/-* hets. **F)** Overview of the dendritic integration simulations. Increasing numbers of proximal and tuft dendrites were activated in a random, but consistent across experiments, sequence with 1 synapse per dendrite. AUC was calculated to identify the change point to supralinearity, conditions were altered, and the procedure was repeated. The first 7 proximal (P) (red) and first 4 tuft (T) (blue) activated dendrites are shown on top of the wt reconstruction used in the dendritic integration simulations, with the numbered dendrites indicating their relative activation sequence. EPSP AUC as a function of different numbers of proximal and tuft dendrites and the corresponding EPSPs under control conditions where only leak current is present **(G)**, leak x2 **(H)**, leak + Ka **(I)**, and leak + Kir **(J)**. Change points were observed where the relation between activated dendrite number and summated EPSP amplitude and duration transitioned from linear to supra-linear. This change point shifted leftward with increasing numbers of activated tuft

dendrites, indicating the facilitation of dendritic integration in the presence of more synaptic inputs. **H)** Doubling the leak conductance, which approximately halved input resistance and approximated the change observed in *Dlg2*+/1 hets once the opposing effects of morphology are taken into account, considerably attenuated EPSP summation and shifted the change point to the right indicating reduced synaptic integration and supra-linearity. **I)** Inclusion of Ka channels into the simulation abolished supra-linear summation. **J)** Inclusion of Kir channels had no effect. **K)** The synaptic NMDAR conductance was increased by a factor 1.4 to approximate the *Dlg2*+/- hets. This shifted the change point to the left but increasing leak conductance reversed the shift in change point. Increasing Ka conductance completely prevented non-linear synaptic summation whereas Kir channels had no effect. **K)** Summary of change point thresholds across simulation conditions with 0 activated tuft dendrites. Summary values depicted as mean ± SEM

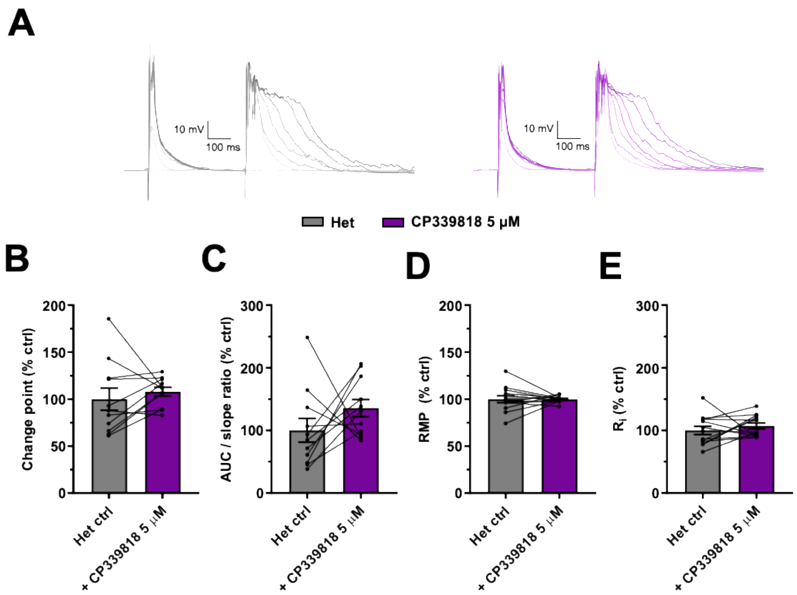

Figure S20 Blocking Kv1.3 and Kv1.4 channels selectively does not affect dendritic integration in the *Dlg2+/-* hets. **A)** Example traces depicting a single EPSP followed by a compound EPSP at increasing stimulation intensities (light to dark) over consecutive recording sweeps before and after CP339818 5 µM. Change point (2-way repeated-measures ANOVA: drug effect: $F_{1, 8} = 2.062$, $P = 0.189$) **(B)**, AUC/slope (2-way repeated-measures ANOVA: drug effect: $F_{1, 8} = 4.553$, $P = 0.065$) **(C)**, resting membrane potential (RMP) (2-way repeated-measures ANOVA: drug effect: $F_{1, 9} = 0.000$, $P = 0.990$) **(D)**, and input resistance (2-way repeated-measures ANOVA: drug effect: $F_{1, 9} = 0.741$, $P = 0.412$) **(E)** as percent of control before and after the after CP339818 5 µM. Hets: 13 cells, 8 animals. Summary values depicted as mean ± SEM. * $P < 0.05$, ** $P < 0.01$, *** $P < 0.001$ (3-way ANOVA between subject effect)

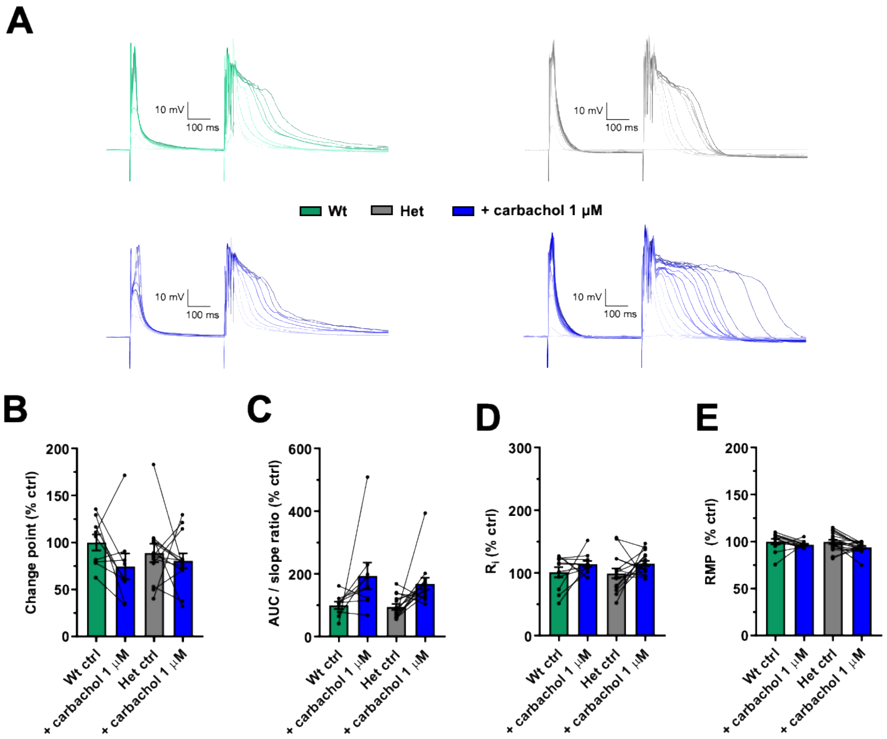

Figure S21 Cholinergic low-dose agonism lowers dendritic integration thresholds in the *Dlg2+/-* hets. **A)** Example traces depicting a single EPSP followed by a compound EPSP at increasing stimulation intensities (light to dark) over consecutive recording sweeps before and after carbachol 1 µM across genotype. Change point (3-way repeated-measures ANOVA: drug effect: $F_{1, 14} = 9.054$, $P = 0.009$. Genotype x drug interaction: $F_{1, 14} = 0.682$, $P = 0.423$) **(B)**, AUC/slope (3-way repeated-measures ANOVA: drug effect: $F_{1, 15} = 28.509$, $P < 0.001$. Genotype x drug interaction: $F_{1, 15} = 0.839$, $P = 0.374$) **(C)**, resting membrane potential (RMP) (3-way repeated-measures ANOVA: drug effect: $F_{1, 21} = 23.469$, $P < 0.001$. Genotype x drug interaction: $F_{1, 21} = 3.254$, $P = 0.086$) **(D)**, and input resistance (3-way repeated-measures ANOVA: drug effect: $F_{1, 21} = 13.203$, $P = 0.002$. Genotype x drug interaction: $F_{1, 21} = 0.001$, $P = 0.974$) **(E)** as percent of control before and after the after carbachol 1 µM across genotype. Hets: 17 cells, 5 animals and wts: 12 cells, 7 animals. Summary values depicted as mean ± SEM. * P < 0.05, ** P < 0.01, *** P < 0.001 (3-way ANOVA between subject effect)

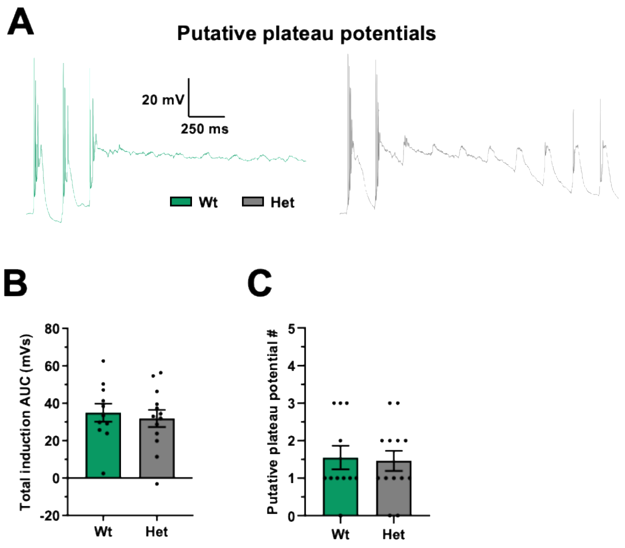

Figure S22 Muscarinic M1 agonism facilitates plateau potential generation in aLTP induction. **A)** example aLTP induction traces showing putative plateau potentials. Total induction area under the curve (AUC) (3-way ANOVA: genotype main effect: $F_{1, 24} = 0.100$, $P = 0.755$) **(B)** and putative plateau potential number (3-way ANOVA: genotype main effect: $F_{1, 24} = 0.002$, $P = 0.963$) **(C)** across genotype. Hets: 13 cells, 7 animals and wts: 11 cells, 6 animals. Summary values depicted as mean ± SEM. * $P < 0.05$, ** $P < 0.01$, *** $P < 0.001$ (3-way ANOVA between subject effect)

References

1. MathWorks. findchangepts [Internet]. [cited 2021 July 23] Available from: https://uk.mathworks.com/help/signal/ref/findchangepts.html
2. Longhair MH, Baker DA, Armstrong JD. Simple Neurite Tracer: open source software for reconstruction, visualization and analysis of neuronal processes. Bioinfo. 2011;27:2453–2454.
3. Ferreira TA, Blackman AV, Oyrer J, Jayabal S, Chung AJ, Watt AJ, Sjöström PJ, van Meyel DJ. Neuronal morphometry directly from bitmap images. Nat Meth. 2014;11:982-984.
4. Carnevale NT, Hines ML, The NEURON book. 2006, Cambridge University Press
5. Migliore M, Hoffman DA, Magee JC, Johnston D. Role of an A-type K+ conductance in the back-propagation of action potentials in the dendrites of hippocampal pyramidal neurons. Comput Neurosci. 1999;7:5-15.
6. Klee R, Ficker E, Heinemann U. Comparison of voltage-dependent potassium currents in rat pyramidal neurons acutely isolated from hippocampal regions CA1 and CA3. Neurophysiol.1995;74:1982-1995.
7. Yim MY, Hanuschkin A, Wolfart J. Intrinsic rescaling of granule cells restores pattern separation ability of a dentate gyrus network model during epileptic hyperexcitability. Hippocamp. 2015;25:297-308.
8. Stegen M, Kirchheim F, Hanuschkin A, Staszewski O, Veh RW, Wolfart J. Adaptive intrinsic plasticity in human dentate gyrus granule cells during temporal lobe epilepsy. Cereb Cort. 2012;22:2087-2101.
9. Wolf JA, Moyer JT, Lazarewicz MT, Contreras D, Benoit-Marand M, O'Donnell P, Finkel LF. NMDA/AMPA ratio impacts state transitions and entrainment to oscillations in a computational model of the nucleus accumbens medium spiny projection neuron. Neurosci. 2005;25:9080-9095.
10. Shah MM, Migliore M, Valencia I, Cooper EC, Brown DA. Functional significance of axonal Kv7 channels in hippocampal pyramidal neurons. Proc Natl Acad Sci USA. 2008;105:7869-7874.
11. McDougal RA, Morse TM, Carnevale T, Marenco L, Wang R, Migliore M, Miller PL, Shepherd GM, Hines ML. Twenty years of ModelDB and beyond: building essential modeling tools for the future of neuroscience. Comput Neurosci 2017;42:1-10.
12. Bloss EB, Cembrowski MS, Karsh B, Colonell J, Fetter RD, Spruston N. Structured dendritic inhibition supports branch-selective integration in CA1 Pyramidal Cells. Neuron. 2016;89:1016-1030.
13. Hemond P, Epstein D, Boley A, Migliore M, Ascoli GA, Jaffe DB. Distinct classes of pyramidal cells exhibit mutually exclusive firing patterns in hippocampal area CA3b. Hippocamp. 2008
14. Baker JL, Perez-Rosello T, Migliore M, Barrionuevo G, Ascoli GA. A computer model of unitary response from associational/commissural and perforant path synapses in hippocampal CA3 pyramidal cells. Comput Neurosci. 2011;31:137-158.
